# Dinuclear Fluoride Single-Bridged Lanthanoid Complexes as Molecule Magnets: Unprecedented Coupling Constant in a Fluoride-Bridged Gadolinium Compound

**DOI:** 10.1021/acs.inorgchem.2c00773

**Published:** 2022-06-23

**Authors:** Julio Corredoira-Vázquez, Cristina González-Barreira, Matilde Fondo, Ana M. García-Deibe, Jesús Sanmartín-Matalobos, Silvia Gómez-Coca, Eliseo Ruiz, Enrique Colacio

**Affiliations:** † Departamento de Química Inorgánica, Facultade de Química, Universidade de Santiago de Compostela, Campus Vida, 15782 Santiago de Compostela, Spain; ‡ Departament de Química Inorgànica i Orgànica, and Institut de Química Teórica i Computacional, Universitat de Barcelona, 08028 Barcelona, Spain; § Departamento de Química Inorgánica, Facultad de Ciencias, Universidad de Granada, Avda Fuentenueva s/n, 18071 Granada, Spain

## Abstract

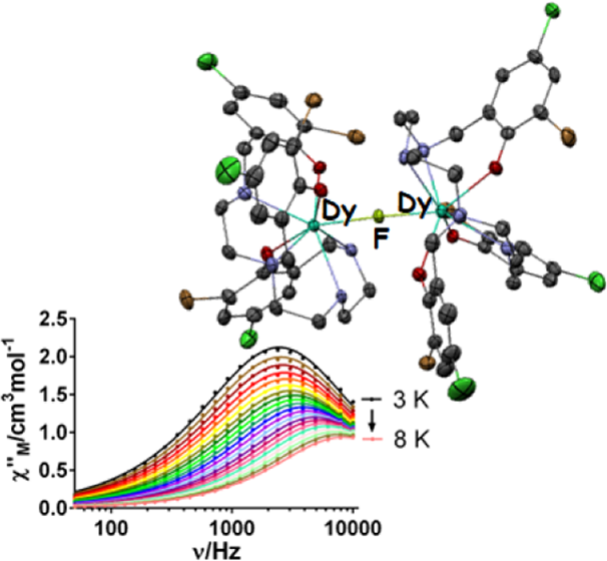

A new synthetic method allows isolating fluoride-bridged complexes Bu_4_N{[M(3NO_2_,5Br-H_3_L^1,1,4^)]_2_(μ-F)} (M = Dy, **1**; M = Ho, **2**; M = Gd, **3**) and Bu_4_N{[Dy(3Br,5Cl-H_3_L^1,2,4^)]_2_(μ-F)}·2H_2_O, **4**·2H_2_O. The crystal structures of **1**·5CH_3_C_6_H_5_,·**2**·2H_2_O·0.75THF, **3**, and **4**·2H_2_O·2THF show that all of them are dinuclear compounds with linear single fluoride bridges and octacoordinated metal centers. Magnetic susceptibility measurements in the temperature range of 2–300 K reveal that the Gd^III^ ions in **3** are weakly antiferromagnetically coupled, and this constitutes the first crystallographically and magnetically analyzed gadolinium complex with a fluoride bridge. Variable-temperature magnetization demonstrates a poor magnetocaloric effect for **3**. Alternating current magnetic measurements for **1**, **2**, and **4**·2H_2_O bring to light that **4**·2H_2_O is an SMM, **1** shows an SMM-like behavior under a magnetic field of 600 Oe, while **2** does not show relaxation of the magnetization even under an applied magnetic field. In spite of this, **2** is the first fluoride-bridged holmium complex magnetically analyzed. DFT and *ab initio* calculations support the experimental magnetic results and show that apparently small structural differences between **1** and **4**·2H_2_O introduce important changes in the dipolar interactions, from antiferromagnetic in **1** to ferromagnetic in **4**·2H_2_O.

## Abstract 2

Dinuclear linear fluoride single-bridged Dy^III^, Ho^III^, and Gd^III^ complexes are systematically obtained from mononuclear aquo-complexes, with the Dy^III^ ones showing slow relaxation of the magnetization and the Gd^III^ one revealing a weak AF coupling through the Gd−F−Gd bridge.

## Introduction

Research in the field of lanthanoid molecular magnets has increased markedly since Rinehart and Long published their electrostatic model that explains how to boost magnetic anisotropy.^1^ According to this theory, oblate ions, like Dy^III^ and Ho^III^, maximize their anisotropy when they are in a crystal field for which the ligand electron density is concentrated above and below the *xy* plane. Therefore, oblate ions in axial fields, like linear, trigonal bipyramidal (tbp), or pentagonal bipyramidal (pbp), should give rise to molecular magnets with improved properties. Consequently, the single-molecule magnet (SMM) with the highest blocking temperature (*T*_B_ = 80 K) reported to date is a pseudo-linear Dy^III^ metallocene,^2^ although a dinuclear mixed-valent dysprosium compound has recently been described that appears to match this *T*_B_.^3^ However, these metallocenes are unstable in air, and compounds with other axial geometries, like pbp, could constitute a good alternative in the search for SMMs with high blocking temperatures. In fact, the *T*_B_ record (36 K) for an air-stable SMM is held by [Dy(bmbpen-F)Br] (H_2_bmbpen-F = *N*,*N*′-bis(5-methyl-2-hydroxybenzyl)-*N*,*N*′-bis(5-fluoro-2-methylpyridyl)ethylene diamine),^4^ a mononuclear complex with Dy^III^ in a pbp environment. Nevertheless, the geometric axis and the anisotropy axis do not always coincide,^5−8^ and accordingly, many complexes with axial geometries have poor magnetic properties.

One way of ensuring axiality in lanthanoid complexes is the selective use of anionic hard donor atoms, like negatively charged oxygen or fluoride. In this way, the best results described to date were found with monodentate oxygen donors,^9−11^ and it seems that oxygen atoms from aliphatic groups provide improved axiality with respect to those from aromatic groups.^9a^ However, many of the SMMs reported with aliphatic negatively charged oxygen atoms, like alcoholates, also seem to be unstable in air.^9^ Besides, it should be noted that the Ln–O bonds should be longer than the Ln–F ones. Therefore, it seems that the use of fluoride as a ligand could be a way of maximizing the anisotropy in lanthanoid metal complexes. This idea is not new, but the synthesis of lanthanoid complexes with the fluoride ligand is, however, challenging because of the tendency of fluoride to form stable and insoluble LnF_3_ compounds.^12^ Thus, the number of magnetically analyzed lanthanoid metal complexes bearing fluoride as a ligand is relatively small.^13−19^ These studies point to the fact that fluoride can create strong axiality even in geometric environments that are not highly axial, such as capped square antiprism.^15^

With these considerations in mind, taking into account that both the synthetic routes and magnetic studies for lanthanoid complexes with fluoride ligands are still scarce, in this work, we report a successful route for the isolation of dinuclear fluoride-bridged dysprosium, holmium, and gadolinium complexes, as well as their magnetic properties.

## Results and Discussion

### Synthesis

**Scheme 1:**
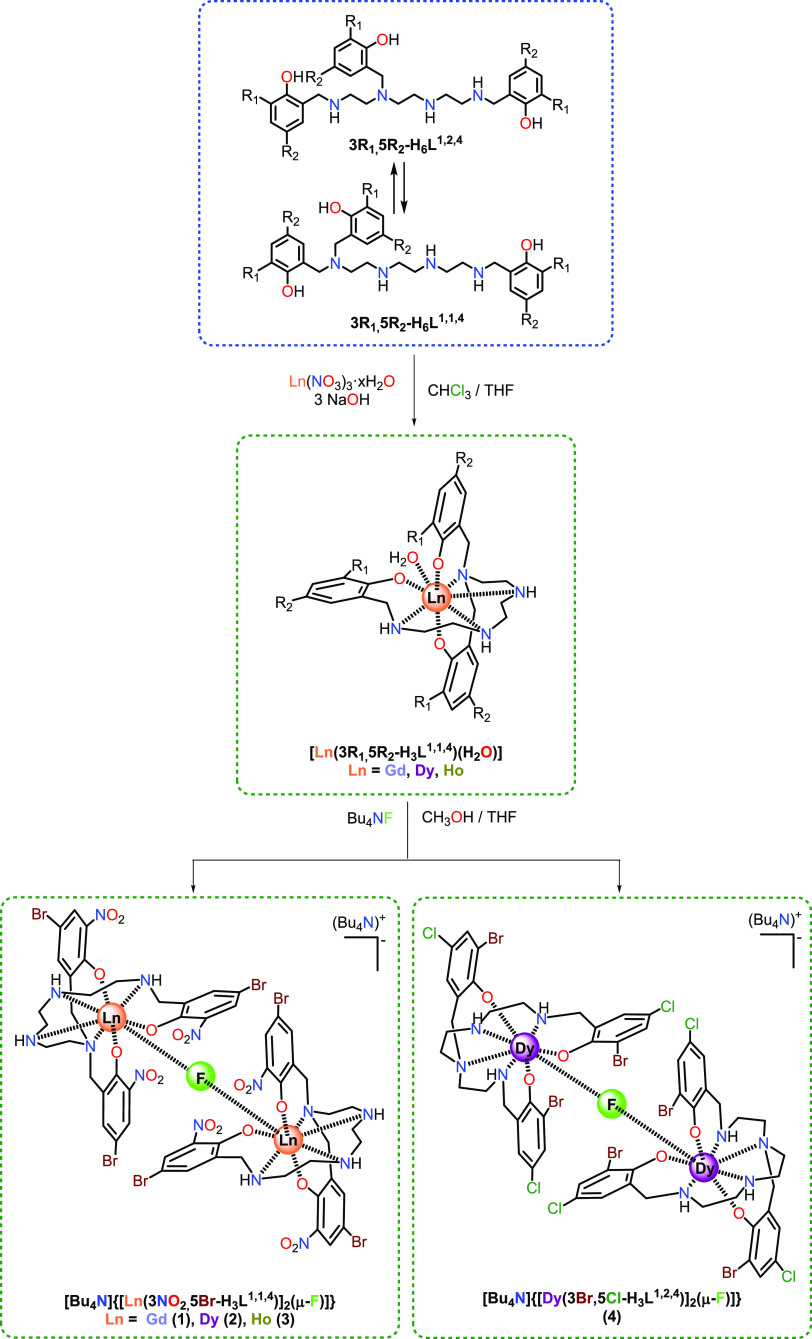
Reaction Scheme for Isolation of the Fluoride-Bridged Complexes

All of the fluoride-bridged complexes **1**–**4**·2H_2_O were obtained by similar processes, by displacement of the water ligand in mononuclear [M(3R_1_,5R_2_-H_3_L^1,1,4^)(H_2_O)] compounds^20^ by a fluoride donor, as summarized in Scheme 1.

However, it is worth noting that, while for fluoride complexes with the 3NO_2_,5Br substituents on the phenol rings (**1**–**3**), the ligand keeps its initial isomeric form (1,1,4),^20^ for the dysprosium complex **4**·2H_2_O, with 3Br,5Cl as substituents on the phenol rings, the aminophenol ligand changes from the initial 1,1,4 isomer^20^ to the final 1,2,4 one. This transformation could seem unexpected, as the equilibrium between the unsubstituted H_6_L^1,2,4^ and H_6_L^1,1,4^ species seems to displace toward the H_6_L^1,1,4^ isomer with increasing pH of the medium.^21,22^ Taking into account that fluoride is a basic anion, the isolation of **4**·2H_2_O with the [3Br,5Cl-H_3_L^1,2,4^]^3–^ aminophenol donor clearly indicates that the substituents on the aromatic ring play a fundamental role in the isomeric equilibrium of this kind of ligand.

The comparison of the experimental powder X-ray diffractogram of the final products with the calculated ones from single X-ray diffraction data (Figure S1) indicates that all of the complexes have been obtained with high purity, without mixtures, and that the collected samples and the solved single crystals are basically the same compounds.

The same dinuclear complexes are isolated when [M(3R_1_,5R_2_-H_3_L^1,1,4^)(H_2_O)] and Bu_4_NF are mixed in a 1:1 or 2:1 molar ratio. However, the yield improves with the 1:1 molar ratio. Nevertheless, pure complexes with terminal fluorides could not be obtained even when this molar ratio was increased to 1:2. Despite this, it represents a systematic method to synthesize lanthanoid complexes with fluoride ligands, a task that is quite arduous, due to the strong tendency of Ln^III^ ions to form LnF_3_ in the presence of fluorides.^12^

Single crystals of all of the complexes could be obtained as detailed below, allowing their single X-ray characterization. However, in spite of the multiple attempts, it was not possible to unequivocally characterize the gadolinium analogues of **4**·2H_2_O.

### X-ray Diffraction Studies

**Figure 1:**
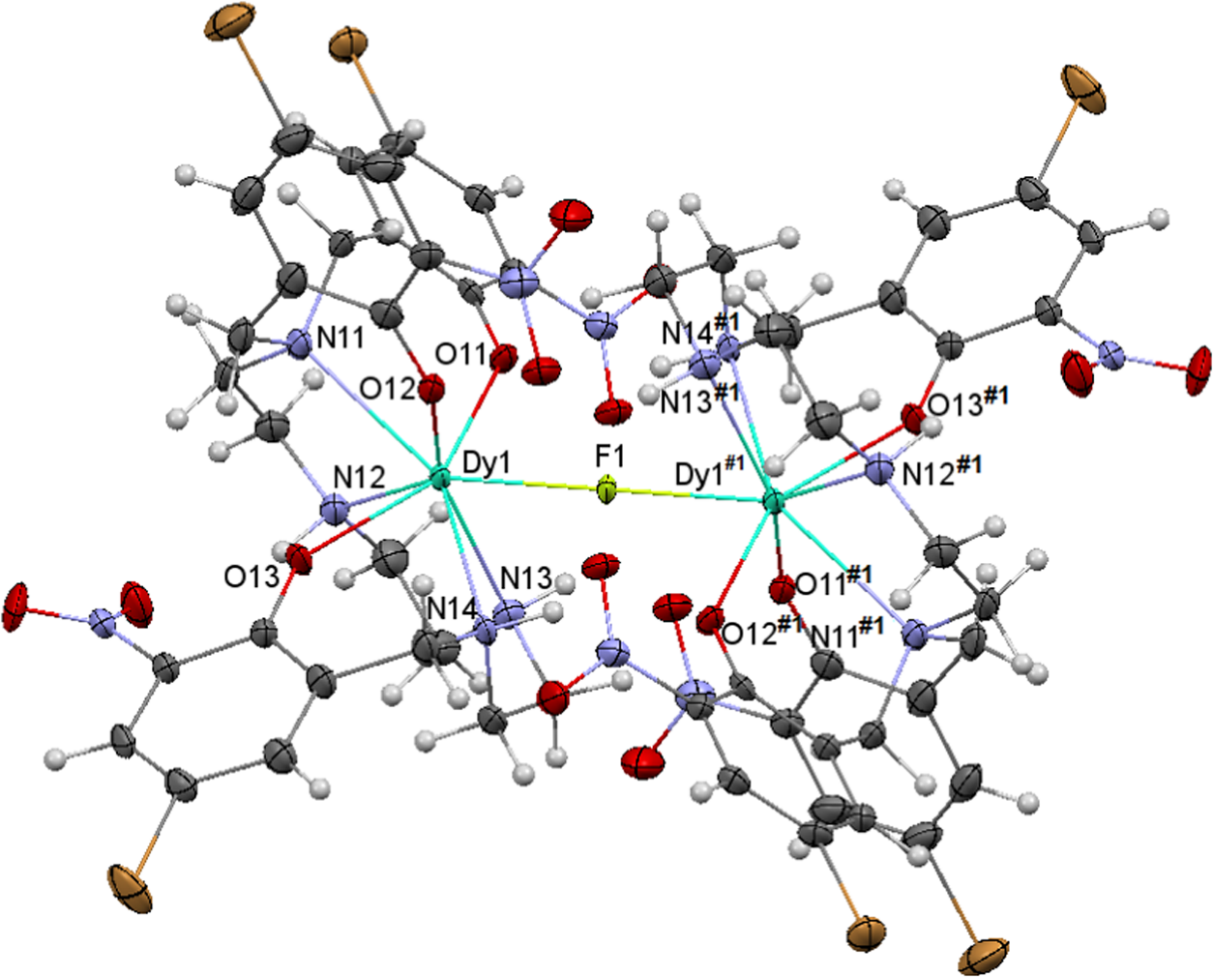
Ellipsoid diagram for the {[Dy(3NO_2_,5Br-H_3_L^1,1,4^)]_2_(μ-F)}^−^ anion in **1.1**.

The crystal structures of Bu_4_N{[Dy(3NO_2_,5Br-H_3_L^1,1,4^)]_2_(μ-F)}·5CH_3_C_6_H_5_ (**1**·5CH_3_C_6_H_5_), Bu_4_N{[Ho(3NO_2_,5-BrH_3_L^1,1,4^)]_2_(μ-F)}·2H_2_O·0.75THF (**2**·2H_2_O·0.75THF), and Bu_4_N{[Gd(3NO_2_,5Br-H_3_L^1,1,4^)]_2_(μ-F)} (**3**) are very similar, and they will be discussed together. The unit cell of the three complexes contains dinuclear {[M(3NO_2_,5Br-H_3_L^1,1,4^)]_2_(μ-F)}^−^ anions (M = Dy, Ho, or Gd) and Bu_4_N^+^ cations, in addition to different solvates. All of the crystals belong to the triclinic group *P*1̅, and in all cases, the asymmetric unit has two chemically equal but crystallographically inequivalent halves of the molecules. Thus, each one of the two Bu_4_N{[M(3NO_2_,5Br-H_3_L^1,1,4^)]_2_(μ-F)} complexes of the unit cell is created by an inversion center, and the crystallographically different molecules will be called **X**.1 and **X**.2 (X = 1 for Dy, X = 2 for Ho, and X = 3 for Gd). Ellipsoid diagrams for the **1**.1, **2**.1, and **3**.1 units are shown in Figures 1, S2, and S3, respectively, and the main bond distances and angles are shown in Table S1. It must be noted that the quality of diffraction data corresponding to the Gd^III^ complex (**3**) was not good enough to be fully anisotropically refined. However, these data allow one to unequivocally know not only the raw structure of the complex but also many structural details with a more than enough accuracy to be presented here.

The structure of all of the {[M(3NO_2_,5Br-H_3_L^1,1,4^)]_2_(μ-F)}^−^ anions in the unit cells (2 per compound) can be understood as two neutral crystallographically equivalent [M(3NO_2_,5Br-H_3_L^1,1,4^)] blocks, joined through a fluoride bridge. Thus, the [M(3NO_2_,5Br-H_3_L^1,1,4^)] unit contains the 1,1,4 isomer of the aminophenol donor, which is the same isomer present in the initial mononuclear complex.^20^ This acts as a trianionic, with all of the phenol oxygen atoms deprotonated and the secondary amine nitrogen atoms protonated. Besides, the ligand behaves as a heptadentate, using all of its oxygen and nitrogen atoms to bind a Ln^3+^ ion. In addition, a fluoride anion bridges the two blocks, thus completing the coordination sphere of the Ln^3+^ centers. This leads to octacoordinated metal ions, with LnN_4_O_3_F cores. Calculations with the SHAPE program^23^ (Table S2) show that the geometry is distorted triangular dodecahedral in all cases. In this polyhedron, the intramolecular Ln···Ln distances are *ca*. 4.5 Å, with a Ln–F–Ln angle of 180° for all of the complexes.

The main Ln–N and Ln–O distances and the angles about the metal centers (Table S1) agree with those expected for complexes with this kind of N,O donor.^20−22,24^ For the Dy^III^ and Gd^III^ compounds, the Ln–F distances are also comparable to the corresponding ones for the scarcely reported single-bridged fluoride dysprosium complexes (Table 1),^13,14^ and, as far as we know, for the only previously crystallographically reported complex with a linear Gd–F–Gd bridge^25^ within CSD.^26^ Besides, and as far as one can tell, there is only one previous homonuclear holmium complex with a fluoride bridge crystallographically characterized,^27^ but, unfortunately, the deposited crystallographic data within CCDC does not allow one to accurately know the geometric parameters (CCDC numbers 115731 and 115732). However, the Ho–F–Ho angle in this polymer is also 180°.

**Table 1:** Comparison of Some Structural and Magnetic Parameters for Dy^III^ Complexes Magnetostructurally Characterized with Fluoride Ligands

compound^a^	Dy–F distance (Å)	Dy–O distance (Å)	Dy–F–Dy angle (deg)	Dy···Dy_intra_ distance (Å)	c.n./geometry^b^	*U*_eff_ K (cm^–1^)/*H*_dc_ (Oe)	references
[DyF(oda)(H_2_O)_3_]*_n_*	2.215(4)/2.249(4)	2.348(4)–2.443(5)	160.26(3)	4.39(7)	8/DD	2.5 (1.7)/0	(13)
[Dy_2_F_2_(oda)_2_(H_2_O)_2_]*_n_*	2.245(2)	2.339(3)–2.428(4)	112.39(3)	3.730(4)	8/n.d.	4.9 (3.4)/0	(13)
[Na_3_Dy_2_(valdien)_2_(μ-F)(μ_3_-F)_2_(Cl)_2_(MeOH)_2_]*_n_*	2.202(3)/2.137(4)/2.209(2)/2.141(4)	2.238(4)–2.278(5)	180.00	4.403(4)/4.418(4)	7/PBPY	49 (34)/0	(14)
[Dy(Tp^py^)F(dioxane)](PF_6_)	2.094(4)	2.533(3)			9/JCSAPR	621.6 (432) (FR)	(15)
						759.7 (528) (SR)/0	
[Dy(Tp^py^)F(pyridine)_2_](PF_6_)	2.0994(16)				9/JCSAPR	483.4 (336)/0	(15)
(Bu_4_N)_8.5_H_1.5_[(PW_11_O_39_)_2_Dy_2_F_2_(H_2_O)_2_]	2.325(6)/2.326(6)	2.264(8)–2.366(7)	112.0(3)	3.8549(10)	7/CTPR	106.5 (74)/0	(16)
[DyLF](CF_3_SO_3_)_2_	2.123(2)				9/MFF	110 (76.5)/0	(17)
[C(NH_2_)_3_]_4_[DyF(piv)_4_](piv)_2_	2.194(2)	2.386(3)–2.557(3)			9/MFF	Raman	(18)
{[Dy(Tp^py^)F(L_c_)]PF_6_}*_n_* (1c)	2.098(4)				9/CSAPR	225.9 (157)/0	(19)
{[Dy(Tp^py^)F(L_o_)]PF_6_}*_n_* (1o)	2.095(4)				9/CSAPR	225.9 (157)/0	(19)
Bu_4_N{[Dy(3-NO_2_,5-Brl-H_3_L^1,1,4^)]_2_(μ-F)}, **1**	2.2764(3)	2.271(3)–2.315(3)	180	4.5528(6)	8/TDD	27.5 (19.1)/600	this work
	2.2717(3)			4.5434(8)			
Bu_4_N{[Dy(3-Br,5-Cl-H_3_L^1,2,4^)]_2_(μ-F)}, **4**	2.1943(5)	2.237(4)–2.331(4)	169.8(2)	4.3709(8)	8/TDD	25.0 (17.4)/0	this work
						35.9 (24.9)/600	

a Solvates omitted. oda, oxidiacetate; valdien, dianion of *N*1,*N*3-bis(3-methoxysalicylidene)diethylenetriamine; Tp^py^, tris(3-(2-pyridyl)pyrazolyl)hydroborate; L, 1,4,7,10-tetrakis(2-pyridylmethyl)-1,4,7,10-tetraaza-cyclododecane; piv, pivalate. n.d., not described.

b c.n., coordination number. Geometry: DD, dodecahedron; PBPY, pentagonal bipyramid; JCSAPR, capped square antiprism; MFF, muffin; CTPR, capped trigonal prism; CSAPR, spherical capped square antiprism; TDD, triangular dodecahedron; BTPR, biaugmented trigonal prism; JBTPR, BTPR J50.

**Figure 2:**
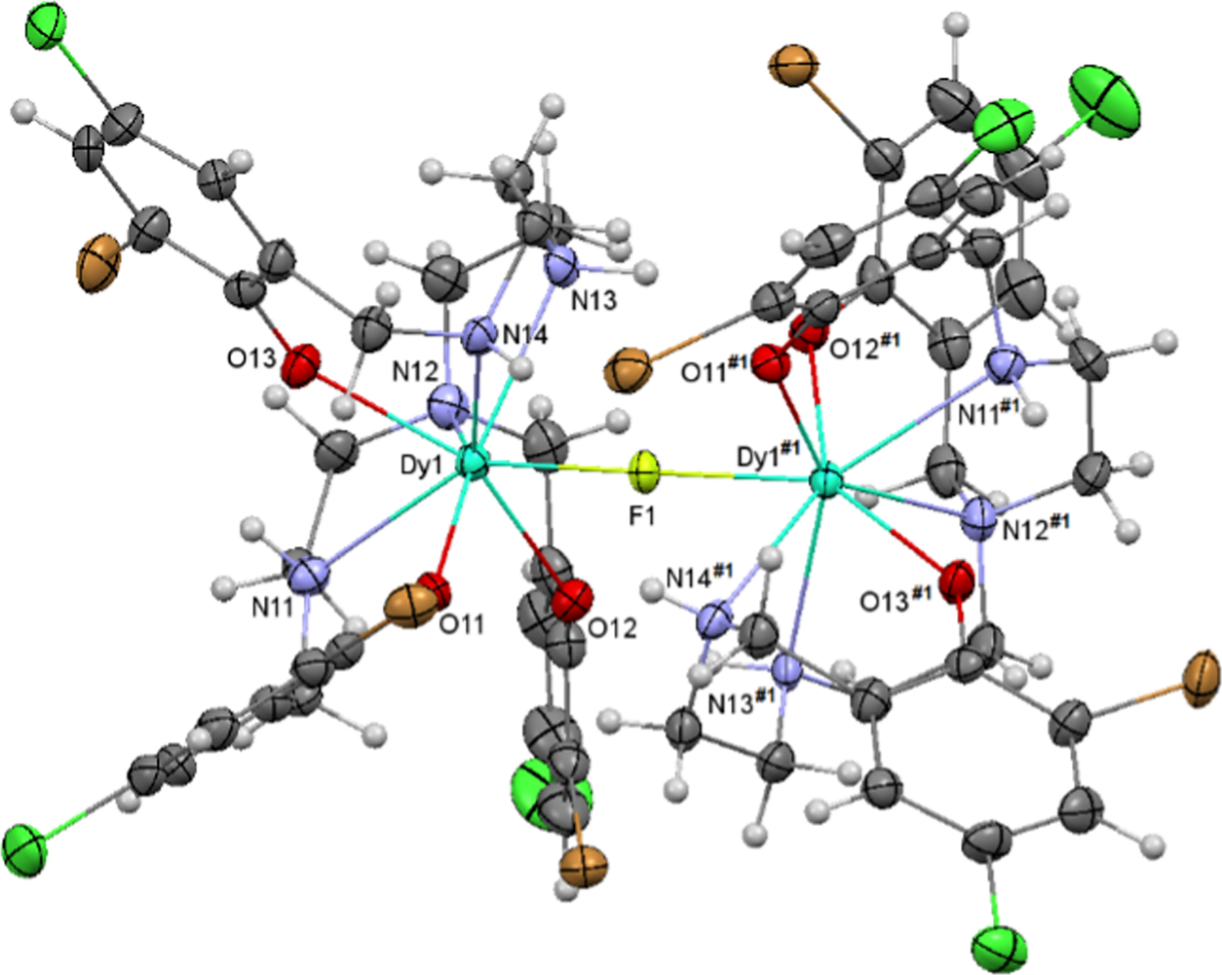
Ellipsoid diagram for the {[Dy(3Br,5Cl-H_3_L^1,2,4^)]_2_(μ-F)}^−^ anion in **4**.

An ellipsoid diagram for Bu_4_N{[Dy(3Br,5Cl-H_3_L^1,2,4^)]_2_(μ-F)}·2H_2_O·2THF (**4**·2H_2_O·2THF) is shown in Figure 2, and the main distances and angles are recorded in Table S1. **4**·2H_2_O·2THF is ionic, as **1**·5CH_3_C_6_H_5_**-3**, and its unit cell contains dinuclear {[M(3Br,5Cl-H_3_L^1,2,4^)]_2_(μ-F)}^−^ anions and Bu_4_N^+^ cations, in addition to THF and water as solvates. The anions have an inversion center, which also makes both [M(3Br,5Cl-H_3_L^1,2,4^)] blocks symmetry-related. In this case, the [M(3Br,5Cl-H_3_L^1,2,4^)] unit contains a different isomer of the aminophenol donor, the 1,2,4 one, which also acts as a trianionic and heptadentate, with all of the amine nitrogen atoms protonated.

The coordination sphere for the Dy^3+^ center is likewise completed by a fluoride ion, as in **1**·5CH_3_C_6_H_5_**-3**. This also leads to octacoordinated dysprosium atoms, in a N_4_O_3_F environment.

Calculations of the degree of distortion of this core with the SHAPE program^23^ indicate that the geometry around the dysprosium atom is also triangular dodecahedral, but in this case, the polyhedron seems to be more distorted toward a biaugmented trigonal prism than in **1**·5CH_3_C_6_H_5_ (Table S2). This highlights the influence of the different isomer of the ligand in the geometry and, hence, in the structural parameters. Accordingly, the Dy–N and Dy–O main distances, and all of the angles about the metal center, agree with those expected for dysprosium complexes with this type of N,O donor,^20,24^ but the DyX–OX1 distances in **4** are significantly shorter than the corresponding ones in **1** (Table S1). In the same way, the Dy–F distance in **4** (*ca*. 2.19 Å) is also within the normal range^12,13^ (Table S1) but notably shorter than in **1** (*ca*. 2.27 Å). Besides, the DyX–OX1 and DyX–OX2 distances in **1** are on the same order of magnitude as the Dy–F ones, while in **4**, the Dy–F distance is appreciably shorter than the Dy–O ones. The same is true for the intramolecular Dy···Dy distance, considerably shorter in **4** (4.3709(8) Å) than in **1** (*ca*. 4.55 Å), with a Dy–F–Dy angle more acute than 180° (169.8(2)°, Table S1) in **4**.

### Magnetic Properties

**Figure 3:**
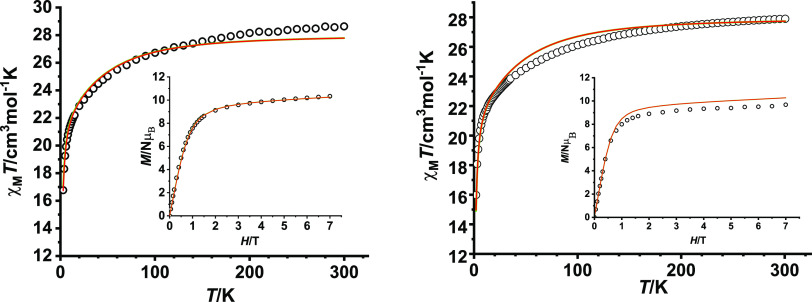
χ_M_*T* vs *T* for: (left) **1**; (right) **4**·2H_2_O. Inset: *M*/Nμ_B_ vs *H* at 3 K. The solid lines represent the theoretical data obtained from *ab initio* calculations.

Direct-current magnetic susceptibility measurements were recorded for **1–4**·2H_2_O as a function of the temperature. The plots of χ_M_*T* vs *T* for complexes of the anisotropic Dy^III^ and Ho^III^ ions (**1**, **2**, and **4**·2H_2_O) are shown in Figures 3 and S4. The χ_M_*T* values at 300 K are 28.6 cm^3^ K mol^–1^ for **1**, 28.5 cm^3^ K mol^–1^ for **2**, and 27.9 cm^3^ K mol^–1^ for **4**·2H_2_O, values that are close to the expected ones for two uncoupled Ln^3+^ ions at room temperature (28.34 cm^3^ K mol^–1^ for Dy_2_, and 28.14 cm^3^ K mol^–1^ for Ho_2_). In all cases, the χ_M_*T* product continuously decreases until 2 K. This drop in the curves can be mainly ascribed to thermal depopulation of the excited *M*_J_ levels, which leads to the presence of significant single-ion anisotropy, and/or to a weak intramolecular antiferromagnetic coupling between the Ln^III^ ions.

The field dependence of the magnetization at 3 K (Figures 3 and S4) shows that the reduced magnetization at the maximum applied field tends to 10.3 Nμ_B_ for **1**, 9.4 Nμ_B_ for **2**, an 9.7 Nμ_B_ for **4**·2H_2_O, values that are far away from the theoretically saturated ones anticipated for two isolated Dy^III^ or Ho^III^ ions (of 20 Nμ_B_ for both ions), thus also suggesting the presence of magnetic anisotropy.

**Figure 4:**
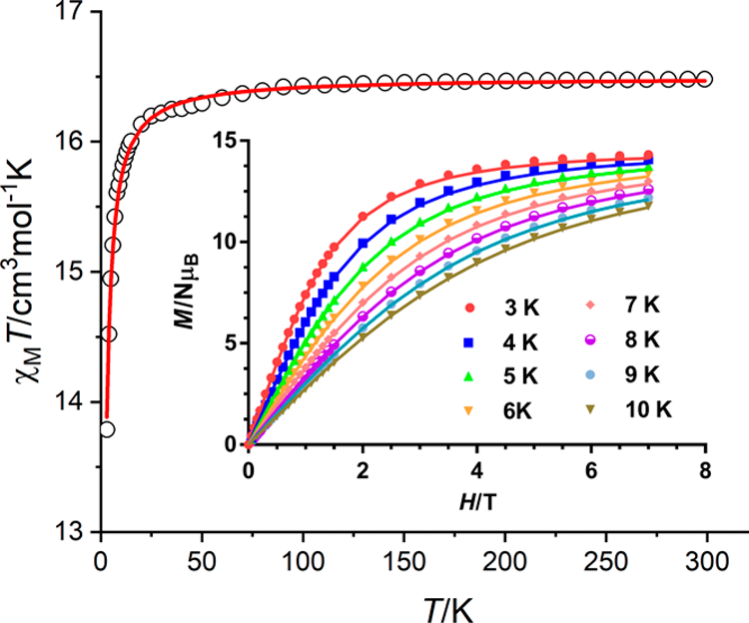
χ_M_*T* vs *T* for **3**. Inset: *M*/Nμ_B_ vs *H* at the indicated temperatures. The solid lines correspond to the best fits.

To probe the strength of the magnetic interaction between lanthanoid ions, the synthesis of Gd^III^ analogues is a common practice due to its isotropic nature. Thus, the Gd^III^ complex **3** was successfully synthesized, as previously mentioned, but the gadolinium compound analogous to **4**·2H_2_O could not be unequivocally characterized. The χ_M_*T* product for **3** is 16.5 cm^3^ K mol^–1^ (Figure 4), a value that is very close to the theoretical one of 15.76 cm^3^ K mol^–1^ for two isolated Gd^III^ ions. This value remains nearly constant until 50 K, and then, it decreases to 13.8 cm^3^ K mol^–1^ at 2 K. This decrease could be mainly due to a small intramolecular antiferromagnetic coupling. In this case, the saturated magnetization at 3 K is 14.0 Nμ_B_, suggesting an *S* = 7 ground state, in agreement with two uncoupled or weakly coupled Gd^III^ ions. The temperature dependence of the χ_M_*T* product and the field dependence of the magnetization at temperatures between 3 and 10 K were simultaneously fitted to the spin Hamiltonian
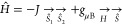
with expected values for *S*_1_ = *S*_2_ = 7/2 and *Ŝ* = *Ŝ*_1_ + *Ŝ*_2_ using the PHI program.^28^ In this equation, the first term accounts for the intramolecular coupling and the second one for the Zeeman effect. The best fit yields the parameters *J* = −0.062(2) cm^–1^ and *g* = 2.047(1).

The value of the coupling constant is very small, and it could be considered meaningless. However, it is not possible to reproduce the curves if this intramolecular AF coupling is not considered. Accordingly, these results agree with a weakly coupled antiferromagnetic system, and they are indicative of very weak antiferromagnetic interactions through the Gd–F–Gd bridge. It must be noted that, as far as we know, studies of fluoride-mediated exchange are hitherto unknown in gadolinium chemistry, and this study unequivocally shows that the exchange in linear Gd–F–Gd bridges is very weak and antiferromagnetic in nature. This is a quite expected result, given that it has been well-documented that because of the shielded nature of the 4f orbitals, the coupling between lanthanoid ions is generally small (<0.1 cm^–1^).^29,30^ The same should also be expected for the coupling constant in **1**, **2**, and **4**·2H_2_O, in view of the χ_M_*T* vs *T* curves, and as the *ab initio* calculations corroborate.

**Figure 5:**
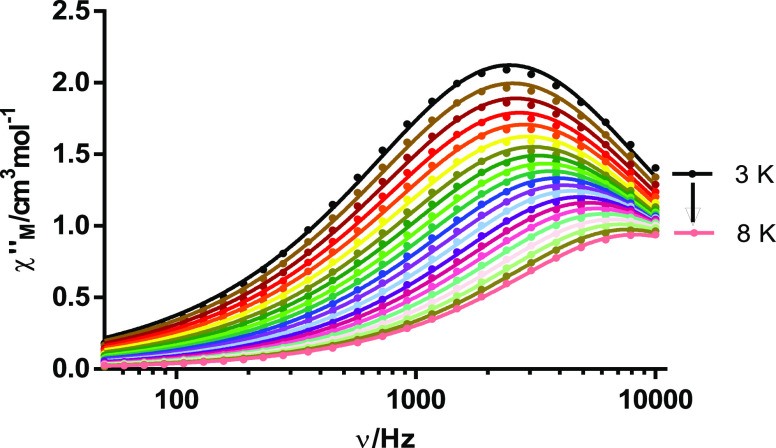
Frequency dependence of χ_M_^″^ for **4**·2H_2_O in a zero dc field at different temperatures.

The dynamic magnetic properties for **1**, **2**, and **4**·2H_2_O were also studied. In a zero dc field, both the in-phase (χ_M_^′^, Figure S5) and out-of-phase (χ_M_^″^, Figures 5 and S5) signals of the ac susceptibility for **4**·2H_2_O feature frequency-dependent phenomena, with peaks for χ_M_^″^ in the temperature range 3–8 K. Thus, **4**·2H_2_O is an SMM. Nevertheless, for **1** and **2**, no peaks for χ_M_^″^ are observed in the absence of a magnetic dc field.

Fitting the Cole–Cole plot to the generalized Debye model for **4**·2H_2_O yields α parameters in the range of 0.16–0.08, which suggest the presence of more than one relaxation process at low temperatures (Figure S6).

**Figure 6:**
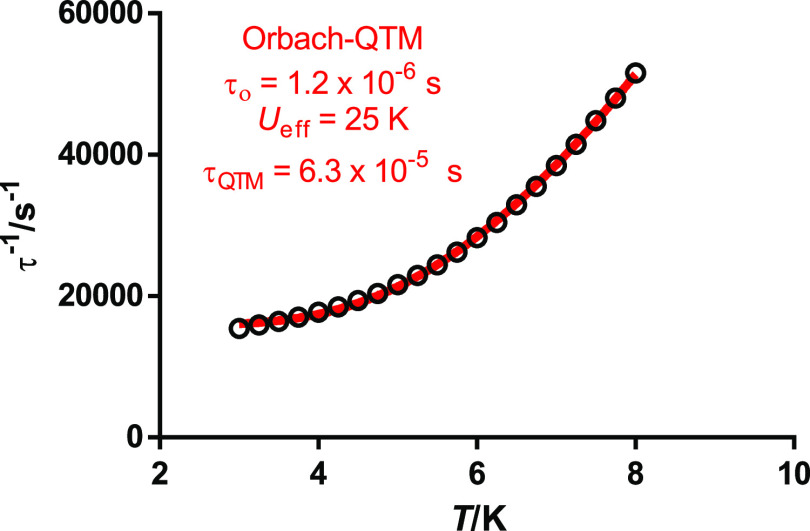
Arrhenius plot for **4**·2H_2_O in zero field. The red solid line accounts for the best fit considering Orbach plus QTM relaxation processes.

The relaxation time and the energy barrier for **4**·2H_2_O were extracted from the Arrhenius plot (Figure 6), whose shape also agrees with several relaxation pathways. In this regard, it should be noticed that χ_M_^″^ does not go to zero below the maxima at low temperatures (Figure S5), which indicates a fast relaxation of the magnetization via a QTM mechanism. Consequently, the Arrhenius plot was fitted including not only all of the possible spin-phonon mechanisms but also the QTM relaxation, according to eq 1.
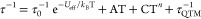
1However, the direct process (second term of the equation) can already be discarded initially since the ac measurements have been collected in the absence of an external magnetic field. The best fit considering the other three processes, individually or grouped, is achieved with Orbach and QTM relaxation. The introduction of the Raman term, which should dominate the low-temperature relaxation regime,^31,32^ does not improve the fit, and leads to overparameterization. Hence, the best fit yields the parameters *U*_eff_ = 25.0 K (17.4 cm^–1^), τ_0_ = 1.2 × 10^–6^, and τ_QTM_ = 6.3 ×10^–5^ s. Accordingly, an appreciable quantum channel is operative in this system. The value of *U*_eff_ is comparable to that obtained for another dysprosium single-bridged fluoride complex.^13^ Nevertheless, it is worth mentioning that **4**·2H_2_O is unique among its class (Table 1), given that, as far as we know, it is the first dinuclear dysprosium single fluoride-bridged complex magnetically characterized (Table 1). The other magnetically analyzed Dy^III^ complexes with single μ-F are polymers,^13,14^ and one of them contains diamagnetic metal ions in its unit cell.^14^ In addition, a dinuclear dysprosium complex with double fluoride angular bridges has been crystallographically and magnetically studied up to now.^16^

**Figure 7:**
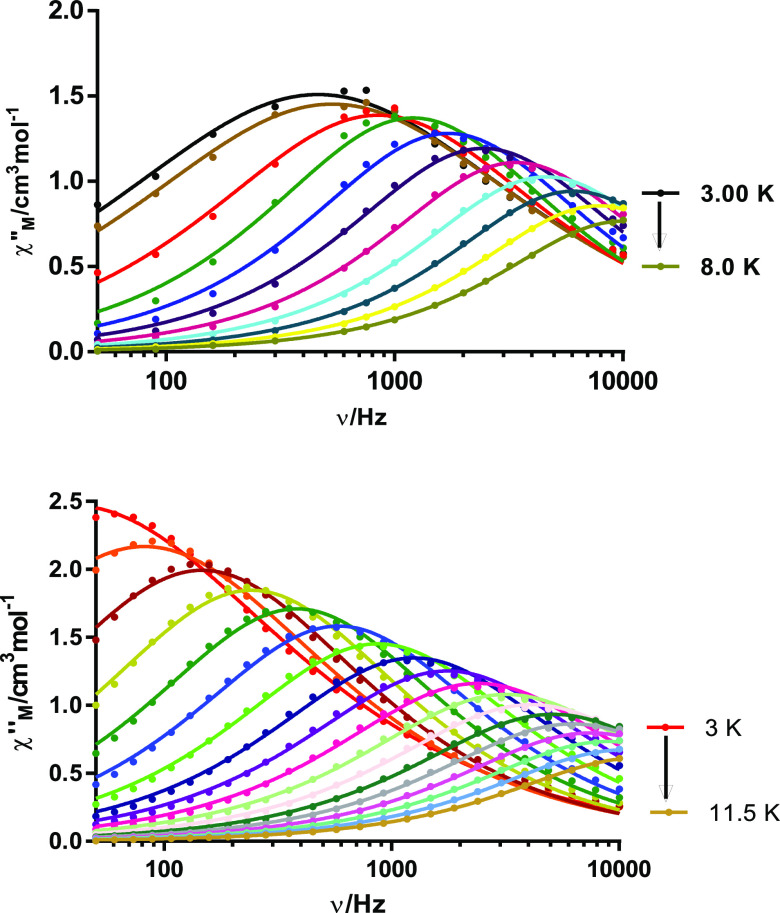
Frequency dependence of χ_M_^″^ for **1** (up) and **4**·2H_2_O (bottom) in *H*_dc_ = 600 Oe.

Given that the fit of the Arrhenius curve indicates that QTM exists for **4**·2H_2_O, and that this QTM can also be the cause for the nonobservation of the SMM behavior in **1** and **2**, attempts were made to eliminate this quantum channel. Thus, new ac measurements were recorded under an external dc optimum field of 600 Oe for all of the complexes (Figure S7). Now, χ_M_^″^ shows frequency- and temperature-dependent peaks for **1** and **4**·2H_2_O (Figures 7 and S8), but not for **2**, which does not present slow relaxation of the magnetization even in the presence of an external field. This can be due to the very small energy barrier for the inversion of the spin, or due to the nonelimination of the QTM.

**Figure 8:**
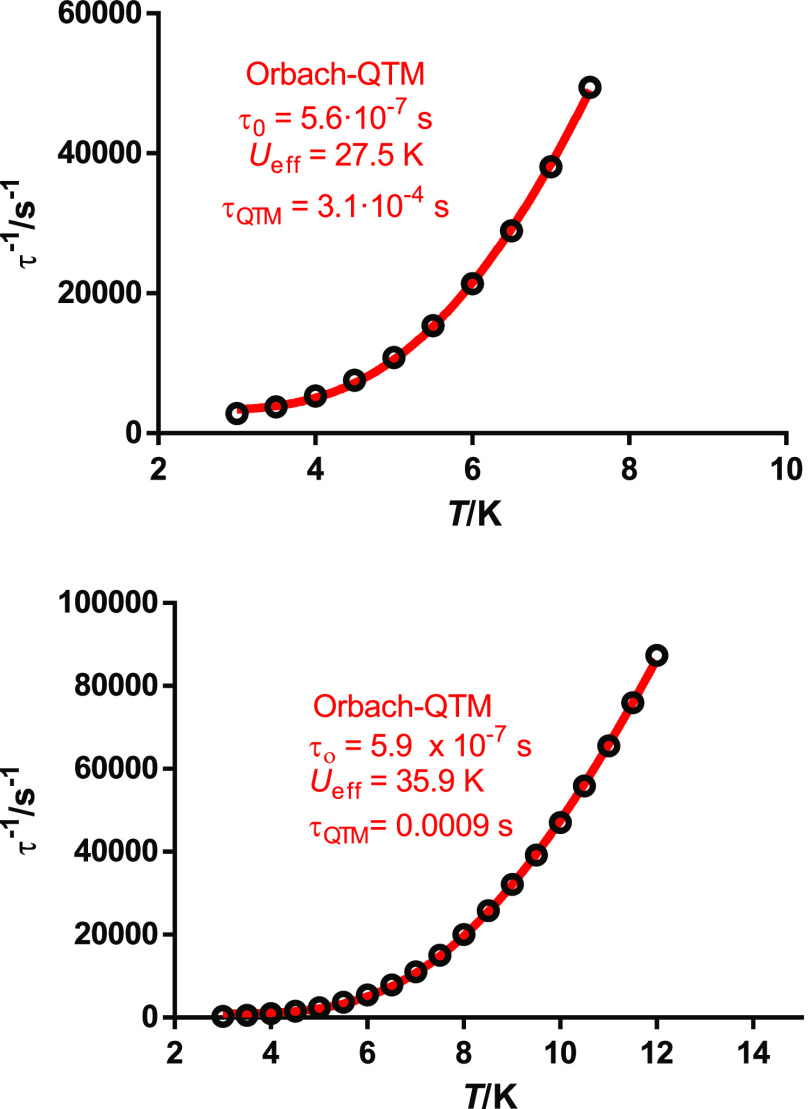
Arrhenius plots for **1** (up) and **4**·2H_2_O (bottom) in *H*_dc_ = 600 Oe. The red solid lines account for the best fit considering Orbach and QTM relaxation processes.

In addition, the χ_M_^″^ vs *T* curves for **1** and **4**·2H_2_O still do not go to zero at low temperature (Figure S8), indicating that the QTM mechanism has not been fully suppressed by the application of the optimum magnetic field. The fit of the Cole–Cole plots for both complexes yields α parameters in the range of 0.13–0.29 for **1** and 0.31–0.11 for **4**·2H_2_O (Figure S9). This, along with the nonlinear shape of the Arrhenius plots (Figure 8), indicates the existence of more than one relaxation process in both cases. Accordingly, the Arrhenius plots for **1** and **4**·2H_2_O were fitted with eq 1, and the best fits taking into account the four processes, individually or grouped, were achieved with only Orbach and QTM relaxation, the latter expected in view of Figure S8. These fits yield the following parameters: *U*_eff_ = 27.5 K (19.1 cm^–1^), τ_0_ = 5.6 × 10^–7^ s, and τ_QTM_ = 0.0003 s for **1**, and *U*_eff_ = 35.9 K (24.9 cm^–1^), τ_0_ = 5.9 × 10^–7^, and τ_QTM_ = 0.0009 s for **4**·2H_2_O. These parameters show that the energy barrier for **4**·2H_2_O is a bit higher than for **1** in the presence of the magnetic field, but that the observed barrier in both cases is small, and on the same order of magnitude, with a larger quantum tunneling for **1**.

### Magneto-Caloric Effect of **3**

Polynuclear Gd^III^ complexes can behave as low-temperature molecular magnetic coolers (MMCs) because they show an enhanced magnetocaloric effect (MCE). This effect depends on the change of magnetic entropy after the application of a magnetic field and can potentially be exploited for cooling applications via adiabatic demagnetization.^33^ In view of this, we decided to evaluate the magnetothermal properties of **3** because of the following reasons: (i) the antiferromagnetic interaction between the Gd^3+^ ions through the fluoride bridging ligand is very feeble and then appropriate for a large magnetocaloric effect (MCE); (ii) the Gd^3+^ ion is rather isotropic due to the lack of orbital contribution; and (iii) Gd^3+^ has the largest single-ion spin (*S* = 7/2), coming from the 4f^7^ electron configuration.

**Figure 9:**
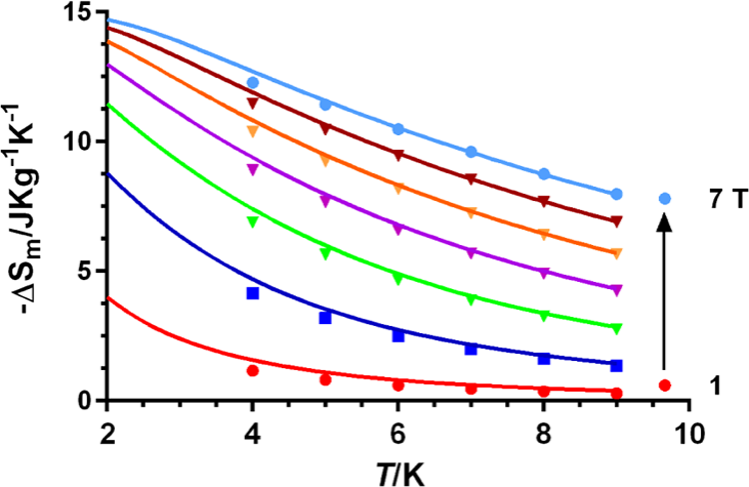
Magnetic entropy changes (−Δ*S*_m_) simulated using *J* = −0.062 cm^–1^ and *g* = 2.047 (solid lines) and calculated from the experimental magnetization data for **3** from 1 to 7 T and temperatures from 4 to 9 K (points).

The magnetic entropy changes (−Δ*S*_m_) that characterize the magnetocaloric properties of **3** can be calculated from the experimental isothermal field-dependent magnetization data (Figure 4) using the Maxwell relation^34^
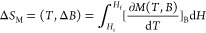
2where *H*_i_ and *H*_f_ are the initial and final applied magnetic fields, respectively. As can be observed in Figure 9, for any applied magnetic field, the values of −Δ*S*_m_ increase with decreasing temperature from 10 to 3 K. The maximum value of −Δ*S*_m_ achieved is 12.27 J kg^–1^ K^–1^ at *T* = 4 K, with an applied field change of Δ*H* = 7 T (Figure 9).

Even though the antiferromagnetic interactions (AF) tend to decrease the values of −Δ*S*_m_ in all cases with regard to the noninteracting systems, when the AF are weak, as in the case of compound **3**, multiple close-in-energy low-lying excited and field-accessible states generate, which would permit an easy polarization of the spin, so that each of these states can contribute to the magnetic entropy of the system, leading to significant −Δ*S*_m_ values at a low magnetic field. We have simulated the MCE for **3** using the magnetic parameters (*g* and *J*) extracted from fitting of the isothermal field dependence of the magnetization and susceptibility data (Figure 9). The simulated −Δ*S*_m_ values are almost equal to those extracted from the Maxwell equations (Figure 9), thus supporting the −Δ*S*_m_ values, and also the coupling constant *J*, extracted from experimental magnetic measurements.

The simulated MCE value at 2 K and 7 T (14.70 J kg^–1^ K^–1^) is only somewhat lower than that calculated for the full magnetic entropy content per mole n*R* ln(2*s*_Gd_ + 1) = 4.16, *R* = 15.41 J kg^–1^ K^–1^ for **3**. Moreover, the extracted −Δ*S*_m_ values are found in the low limit of the MCE observed for other Gd_2_ complexes,^33c^ which can mainly be attributed to the low magnetic density of **3** (n° Gd^3+^/MW), because the increase in magnetic density produces an increase of MCE. This result again highlights the essential role of magnetic density on the magnitude of the MCE.

### *Ab Initio* Calculations

To gain more insights into the magnetic properties of the studied compounds, *ab initio* calculations were performed for dysprosium and the holmium complexes based on their single-crystal X-ray structures (see Computational Details) without solvates. This decision was based on our own experience, which shows that solvates do not have a significant influence on the calculations in this kind of complex.^8,20,24c^ In addition, for **1** and **2**, calculations were performed for the crystallographically different **X**.1 and **X**.2 (X = 1 or 2) complexes present in the unit cell. Fragment calculations were made for both halves, but due to symmetry reasons, only the results of one Ln for each complex are shown and have been discussed (see Tables S3–S5).

The obtained *g*-factors for **1** and **4** are collected in Table 2. Both complexes show a large axial character of the ground state but with nonzero *g_x_* and *g_y_* components, indicating a relatively non-negligible QTM, as is experimentally observed.

**Table 2:** Calculated *g* Components for **1.1**, **1.2**, and **4** for the Ground and First Excited States at the CASSCF Level^a^

	1.1		1.2		4	
compound	GS	1st ES	GS	1st ES	GS	1st ES
*g_x_*	0.137	0.802	0.111	1.134	0.050	0.285
*g_y_*	0.314	6.207	0.269	2.041	0.140	0.518
*g_z_*	19.097	10.942	19.222	15.470	18.909	14.842
θ (deg)	58.0		59.1		28.1	
γ (deg)	139.8		34.4		2.5	

a θ, angle between the *g_z_* vector and the vector connecting both Dy^III^ in the dinuclear molecule; γ, angle between the *g_z_* vectors of the ground and first excited states.

**Figure 10:**
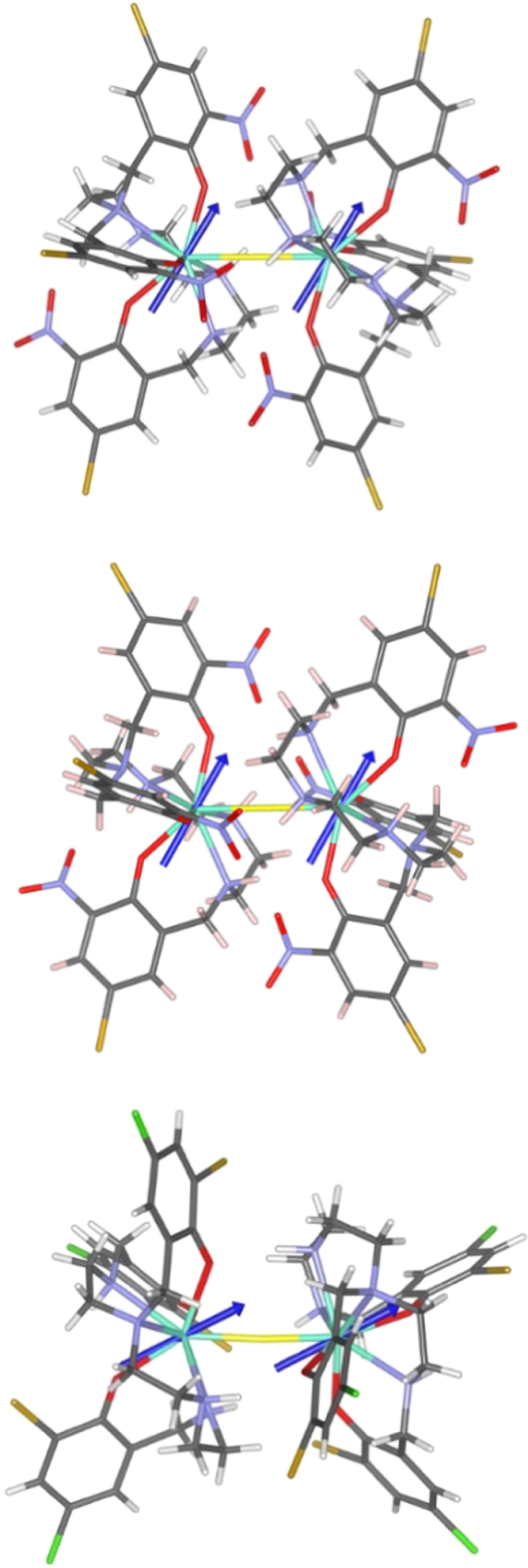
Molecular structures of **1.1** (up), **1.2** (middle), and **4** (bottom) showing the *g_z_* calculated directions of the *g* components of the Dy^III^ centers in the ground state.

The direction of the easy axis will depend on the coordinated ligands. For the Dy^III^ compounds, the oblate shape of the electron density of the Dy^III^ center will be accommodated between the ligands surrounding the metal in the best possible way to reduce the electronic repulsion. The coordinated ligands have three monoanionic phenolic oxygen atoms (OX1, OX2, and OX3 in both compounds), and the Dy^III^ centers are bridged by the fluoride anion. Three of those negatively charged donors (two phenolic oxygen atoms, OX1 and OX3, and the fluoride) are in the same plane, and the oblate electron density of the Dy^III^ center will avoid that plane from being perpendicular to it. Consequently, the directions of the *g_z_* component will be in that plane in both compounds. In both cases, there is a phenolic oxygen atom close to the fluoride (OX1, O–Dy–F angle of about 74°) and another further away (OX3, O–Dy–F angle of about 142°). To minimize repulsions, the oblate electron density of the Dy^III^ center will be allocated between the phenolic oxygen OX1 and fluoride atoms, and the second phenolic oxygen, OX3. In the case of **4**, the Dy–F distance is the shortest one, and the *g_z_* component is located closer to the fluoride, while in **1**, the distances Dy–O and Dy–F are very similar, as previously discussed, and the *g_z_* component is located closer to the phenolic oxygen OX1 (see Figure 10). As a result, the angle between the *g_z_* vector and the vector connecting both Dy^III^ ions (θ) in the dinuclear molecule is quite different (see Table 2), which will affect the dipolar exchange coupling, *vide infra*.

The analysis of the lowest energy states before inclusion of the spin–orbit effect, collected in Table S3, shows that the first excited state is very close in energy in all of the cases (between 14 and 20.6 cm^–1^), while the second excited state is further away (between 106.3 and 146 cm^–1^). Some of us previously noticed that the value of (*E*_2_ – *E*_1_)/*E*_1_ is a figure of merit of the axiality in mononuclear Dy compounds.^35^ Herein, the ratio is larger in compound **4**. This usually results in a large axiality because the two states that are very close in energy, the ground and first excited states, are very axial, while the second excited state has a less axial character. However, as can be observed in the *g_i_* components of the *g* factor in Table 2, here, the ground state is quite axial in both **1** and **4** (although in **4** the *g_x_* and *g_y_* components are smaller), but the excited states for **4** are more axial than for **1**. Nevertheless, the analysis of the energies of the lowest KDs after the inclusion of the SOC states shows a large energy difference between the ground and the first excited KDs (around 100 cm^–1^) in all of the cases (Table S4), although the next KD (the second excited state) is very close in energy to the first state for compound **1.1**.

**Figure 11:**
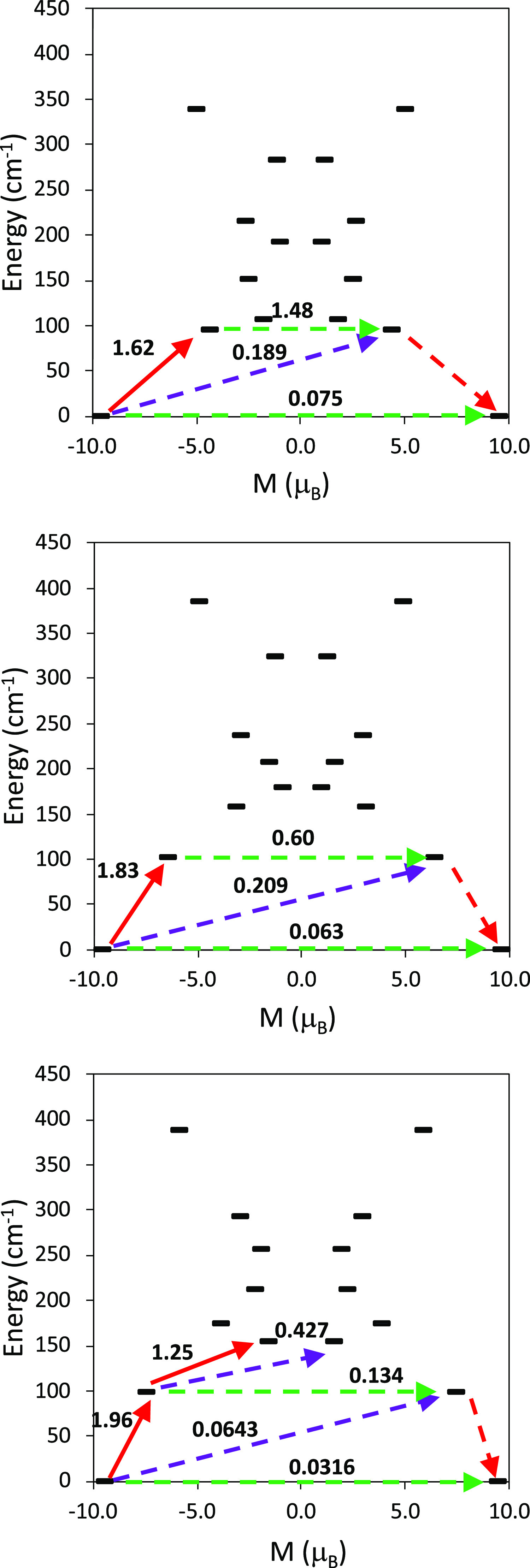
States’ energies as a function of their average magnetic moment, *M*, along the main anisotropy axis for the individual fragments of compounds **1.1** (top), **1.2** (middle), and **4** (bottom). The dashed green arrows correspond to the quantum tunneling mechanism of the ground or excited states, and the dashed purple arrow shows the hypothetical Orbach relaxation process. The solid red arrow indicates the transition between the ground and excited Kramers doublets, and the dashed red arrow indicates the excitation pathway to the ground state with the reversed spin. The values close to the arrows indicate the matrix elements of the transition magnetic moments (above 0.1, an efficient spin relaxation mechanism is expected).

When looking at the transition probabilities between the states of the individual fragments (Figure 11), it can also be seen that in all of the complexes the quantum tunnel probability in the ground state is lower than 0.1, and that the probabilities are in general smaller for **4** than for **1**, which is in concordance with the small values of *g_x_* and *g_y_* in Table 2. In the case of compound **4**, the Orbach relaxation through the first excited state has a probability lower than 0.1, which is in concordance also with the close-to-parallel orientation of the easy axis in both the ground and first excited states (see Table 2). Thus, the expected mechanism would be thermally assisted QTM through the first KD (*U*_eff_*ca*. 100 cm^–1^), but the Orbach process through the second excited state is also highly probable (*U*_eff_*ca*. 156 cm^–1^), especially if an external field is applied.

For **1**, the predominant mechanism seems to be Orbach relaxation through the first excited state in both cases, with an energy of *ca*. 96–102 cm^–1^. Besides, although the mechanism seems to be the same in **1**.1 and **1**.2, several differences can be observed. For **1**.1 the second excited state is closer in energy to the first one, and it has a larger tunneling probability in the first excited state than that observed for **1**.2, which might be related to the larger axiality of the first excited state in **1**.2 (Table 2). The obtained values, although quite far from the calculated ones from magnetic measurements, allow explaining the experimental results. Accordingly, **1** is not an SMM, and this can be related to the more feasible QTM mechanism for **1** compared to **4**. This agrees with the fact that slow relaxation of the magnetization appears experimentally for **1** only in the presence of a magnetic field, thus revealing the strong QTM for **1**. The best magnetic behavior of **4**, which is an SMM, compared to **1**, is also justified by the *ab initio* calculations, given that they clearly demonstrate that the ground state and, specially, the first excited state are more axial (smaller *g_x_* and *g_y_* values) in **4** than in **1** and that the QTM is less probable in **4**.

Nevertheless, the experimentally calculated *U*_eff_ value for **4** (24.9 cm^–1^ in *H*_dc_ = 600 Oe) differs significantly from the theoretical one. The same occurs for **1** (19.1 cm^–1^ in *H*_dc_ = 600 Oe). This difference could perhaps be ascribed to some effects related to magnetic interactions, which possibly are not considered in the *ab initio* calculations, and also to the presence of other competing relaxation mechanisms. In spite of this, these studies show the same experimental trend, and are in agreement with the fact that the compound with the 3Br,5Cl substituents has better magnetic properties than the compound with the 3NO_2_,5Br substituents, as we have already published.^20^ However, in this case, the difference in the magnetic behavior seems not to be attributable to the greater electrophilic character of the substituents in **1**, but rather seems to be related to structural changes resulting from the presence of the 1,1,2 isomer of the ligand in **1** and the 1,2,4 one in **4**.

The magnetic coupling interaction between Dy^III^ centers in both complexes has been also studied by means of POLY_ANISO software and the implemented Lines model. Both magnetization and susceptibility curves were fitted simultaneously with and without the inclusion of the dipolar magnetic coupling, and we found a reasonable agreement with the experimental susceptibility and magnetization curves (Figure 3). For compound **4**, if the magnetic dipolar coupling is not included, the obtained coupling constant is −0.14 cm^–1^, while if we include the dipolar coupling, then the coupling constant value is −0.29 cm^–1^, showing the ferromagnetic nature of the dipolar coupling (+0.15 cm^–1^). In the case of **1**, however, the coupling is smaller, and it goes from −0.10 (−0.106) cm^–1^ to −0.094 (−0.094) cm^–1^ for **1**.1 (**1**.2) when the dipolar coupling is considered. In this case, this dipolar coupling is antiferromagnetic and very small (*ca*. −0.01 cm^–1^). The magnetic dipolar coupling mainly depends on the magnetic moments of the interacting centers, their relative orientation, and the distance between them. Their expression can be simplified into eq 3 in the case of two parallel interacting magnetic moments, which is the case in both dysprosium compounds.
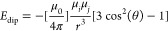
3In eq 3, it can be seen how it depends on the angle between the magnetic moment and the vector connecting both magnetic centers. When this angle is smaller than 54.75°, the coupling is ferromagnetic, while when it is larger, it is antiferromagnetic. In addition, the coupling is maximized when the magnetic moment and the vector connecting both magnetic centers are parallel or perpendicular. As can be seen in Figure 10 and Table 2, for compound **4**, the angle is smaller (*ca*. 28°) and for **1** it is slightly larger (*ca*. 58–59°) than 54.75°, which agrees with the obtained ferromagnetic dipolar coupling for **4**, and the very small and antiferromagnetic coupling for **1**. Additionally, the exchange coupling through the fluoride bridge is relatively weak and antiferromagnetic in both cases, −0.094 cm^–1^ for **1** and −0.29 cm^–1^ for **4**. The weak exchange coupling is expected for lanthanoid ions, although herein, larger coupling is observed for Dy in comparison to Gd ions, especially in compound **4**, which might be related to the differences in the bridge. Due to the small value of the exchange interaction, the consideration of the exchange/dipolar interaction on the low-lying spectrum gives rise to a similar energy barrier (Tables S6–S8). In the low-lying spectrum, there are four states very low in energy (less than 2 K) due to the possible ferro- and antiferromagnetically coupling states of the ground KDs, which will be populated at the temperature of the experiment. At an energy closer to the first excited KD in the individual fragments, eight states would be found resulting from the coupling of the ground KDs and the first excited KDs. The transition probabilities also found in this scheme show that the relaxation will be through states at the same energy differences as those found for the individual fragments.

Accordingly, these results clearly show that small structural changes can lead to very significant changes in the dipolar interactions and, hence, in the magnetic behavior of metal complexes although the calculated energy barriers in these cases are not affected.

In the case of compound **2**, the lowest energy states can be described as ^5^I_8_ ground multiplets of the Ho^III^ center, which are split in a range of 250 cm^–1^ (Table S5), which is similar to other Ho compounds.^24c,36^ The small energy difference between the ground state and the first excited states may account for the absence of slow relaxation even in the presence of an external field, to which the large QTM in this non-Kramer system can also contribute. The magnetic coupling interaction between Ho^III^ centers has been also studied with the same procedure employed for the Dy analogue (**1**). In this case, although the calculations reproduce the curve worse than for the dysprosium compounds, and there are several fitting possibilities, the experimental values can be reasonably simulated with a small *J* value of −0.1 cm^–1^ (Figure S4), similar to that of compound **1**.

In addition, DFT calculations for the gadolinium complex **3**, based on its single-crystal X-ray structure, were also performed (see the Computational Details). The calculated *J* value for this dinuclear Gd_2_ complex (*Ĥ* = −*JŜ*_1_*Ŝ*_2_) is −0.028 cm^–1^. Despite the tiny value, it is on the same order of magnitude as the experimental one (−0.062 cm^–1^), and the antiferromagnetic character is well reproduced. This calculated value corresponds to the exchange term, and experimentally, such a contribution is also mixed with dipolar interactions, which are expected to be smaller. Thus, these calculations also validate the experimental small antiferromagnetic exchange mediated by the linear fluoride bridge in the gadolinium complex **3**.

## Conclusions

This work describes a new synthetic method for isolating linear fluoride single-bridged dinuclear complexes from mono-aquo mononuclear precursors. All of the four complexes described herein contribute to an increase in the scarce number of fluoride-lanthanoid coordination compounds crystallographically and magnetically studied. Thus, the Gd^III^ complex **3** is, as far as we know, the first crystallographically and magnetically analyzed gadolinium complex with a fluoride bridge. The magnetic study shows that the coupling constant through a linear Gd–F–Gd bridge is small and antiferromagnetic in nature, and this was also sustained by theoretical calculations. **2** is the first fluoride holmiun complex to be magnetically analyzed and, to the best of our knowledge, the second one of being crystallographically characterized. Nevertheless, **2** does not show slow relaxation of the magnetization even in the presence of a magnetic field, while dysprosium complexes **1** and **4**·2H_2_O do. However, while **4**·2H_2_O is an SMM, **1** only presents an SMM-like behavior under a magnetic field of 600 Oe. The *U*_eff_ barriers for **1** and **4**·2H_2_O are on the same order of magnitude, being a bit smaller for **1** than for **4**·2H_2_O. This tendency is also corroborated by *ab initio* studies, which show the less axial character, and the most probable QTM for **1**, as well as justify the absence of SMM behavior for **2**. These calculations also highlight the different dipolar interactions in **1** (antiferromagnetic) and **4**·2H_2_O (ferromagnetic), and further support the overall antiferromagnetic nature of the coupling constant in **3**.

## Experimental Section

### Materials and General Methods

All chemical reagents were purchased from commercial sources and used as received without further purification. Elemental analyses of C, H, and N were performed on a Themoscientific Flash Smart analyzer. The ligands were prepared as previously reported.^20^

### Synthesis of the Complexes

#### Mononuclear Complexes

The mononuclear precursors [Dy(3Br,5Cl-H_3_L^1,1,4^)(H_2_O)]·0.25MeOH and [Dy(3NO_2_,5Br-H_3_L^1,1,4^)(H_2_O)] were obtained as previously reported by us,^20^ while [Ho(3NO_2_,5Br-H_3_L^1,1,4^)(H_2_O)] and [Gd(3NO_2_,5Br-H_3_L^1,1,4^)(H_2_O)] are new, and were prepared in a similar way, exemplified by the isolation of [Ho(3NO_2_,5Br-H_3_L^1,1,4^)(H_2_O)]: To a solution of 3NO_2_,5Br-H_3_L^1,1,4^ (0.100 g, 0.120 mmol) in acetonitrile/chloroform (20/15 mL), triethylamine (0.036 g, 0.359 mmol) is added. Then, a solution of holmium nitrate pentahydrate (0.053 g, 0.120 mmol) in acetonitrile (15 mL) is added to the ligand solution, and the mixture is stirred at room temperature for 24 h. The solution is filtered to avoid any possible impurity, and then, it is concentrated in a rotaevaporator up to 15 mL. The precipitated solid is filtered and dried in an oven for 4 h. Yield: 0.056 g (47%). M.W.: 1013.14 g/mol. Anal. calcd for C_27_H_26_Br_3_HoN_7_O_10_ (%): C 32.01, N 9.68, H 2.59. Found: C 32.59, N 9.85, H 2.43.

#### [Gd(3NO_2_,5Br-H_3_L^1,1,4^)(H_2_O)]

3NO_2_,5Br-H_3_L^1,1,4^ (0.200 g, 0.240 mmol) in methanol (10 mL)/chloroform (10 mL), NaOH solution (0.029 g, 0.717 mmol) in methanol (5 mL); Gd(NO_3_)_3_·6H_2_O (0.108 g, 0.239 mmol) in methanol (5 mL). Yield: 0.163 g (68%). M.W.: 1008.52 g/mol. Anal. calcd for C_27_H_29_Br_3_GdN_7_O_10_: C 32.15, N 9.72, H 2.90. Found: C 31.99, N 9.75, H 3.00.

#### Fluoride-Bridged Complexes

All of the dinuclear fluoride-bridged complexes were obtained in a similar way, from the mononuclear complexes [Dy(3NO_2_,5Br-H_3_L^1,1,4^)(H_2_O)], [Ho(3NO_2_,5Br-H_3_L^1,1,4^)(H_2_O)], [Gd(3NO_2_,5Br-H_3_L^1,1,4^)(H_2_O)], and [Dy(3Br,5Cl-H_3_L^1,1,4^)(H_2_O)]·0.25MeOH. Their isolation is exemplified by the synthesis of Bu_4_N{[Dy(3NO_2_,5Br-H_3_L^1,1,4^)]_2_(μ-F)} (**1**): To a MeOH/THF (25/25 mL) solution of [Dy(3NO_2_,5Br-H_3_L^1,1,4^)(H_2_O)] (0.086 g, 0.085 mmol), Bu_4_NF (0.027 g, 0.085 mmol) is added. The mixture is stirred overnight, and a solution is obtained. The solution is filtered to eliminate any impurity and left to slowly evaporate. After 2 days, an orange powder precipitates, which is isolated by filtration. Recrystallization of the orange powder in toluene gives rise to single crystals of **1**·5CH_3_C_6_H_5_. The crystals lose the toluene solvate on drying. Yield: 0.072 g (38%). M.W.: 2252.98 g/mol. Anal. calcd for C_70_H_90_Br_6_Dy_2_N_15_O_18_F: C 37.32, N 9.33, H 4.03%. Found: C 37.15, N 9.18, H 4.08.

#### Bu_4_N{[Ho(3NO_2_,5Br-H_3_L^1,1,4^)]_2_(μ-F)} (**2**)

[Ho(3NO_2_,5Br-H_3_L^1,1,4^)(H_2_O)] (0.174 g, 0.171 mmol) in THF (25 mL); Bu_4_NF (0.055 g, 0.171 mmol). Slow evaporation of the obtained solution yields single crystals of the **2**·2H_2_O·0.75THF, suitable for single X-ray diffraction studies. The crystals lose the solvates on drying, to produce 2. Yield: 0.123 g (32%). M.W.: 2257.84 g/mol. Anal. Calcd for C_70_H_90_Br_6_Ho_2_N_15_O_18_F: C 37.24, N 9.31, H 4.02%. Found: C 37.10, N 9.42, H 4.33%.

#### Bu_4_N{[Gd(3NO_2_,5Br-H_3_L^1,1,4^)]_2_(μ-F)} (**3**)

[Gd(3NO_2_,5Br-H_3_L^1,1,4^)(H_2_O)] (0.193 g, 0.191 mmol) in MeOH/THF (25/25 mL) THF (25 mL). Bu_4_NF (0.051 g, 0.191 mmol). Single crystals of **3**, suitable for single X-ray diffraction studies, are obtained from the mother liquors. Yield: 0.160 g (37%). M.W.: 2242.48 g/mol. Anal. calcd for C_70_H_90_Br_6_Gd_2_N_15_O_18_F: C 37.49, N 9.37, H 4.05%. Found: C 37.02, N 9.17, H 4.01%.

#### Bu_4_N{[Dy(3Br,5Cl-H_3_L^1,2,4^)]_2_(μ-F)}·2H_2_O (**4**·2H_2_O)

[Dy(3Br,5Cl-H_6_L^1,1,4^)(H_2_O)]·0.25MeOH (0.148 g, 0.149 mmol), Bu_4_NF (0.039 g, 0.149 mmol) in MeOH/THF (25/25 mL). Slow evaporation of the obtained solution directly renders single crystals of **4**·2H_2_O·2THF, suitable for X-ray diffraction studies, which loses the THF solvate to give rise to **4**·2H_2_O. Yield: 0.116 g (35%). M.W.: 2225.60. Anal. calcd for C_70_H_94_Dy_2_Br_6_Cl_6_N_9_O_8_: C 37.77, N 5.66, H 4.26%: Found: C 37.51, N 5.53, H 4.09%.

### Single X-ray Diffraction Studies

Crystal data and details of refinement are given in Table S9. The single crystals of **1·**5CH_3_C_6_H_5_, **2**·2H_2_O·0.75THF, **3**, and **4**·2H_2_O·2THF could be obtained as detailed above. Data were collected at 100 K on a Bruker D8 VENTURE PHOTON III-14 diffractometer, employing graphite monochromatized Mo-Kα (λ = 0.71073 Å) radiation. Multiscan absorption corrections were applied using the SADABS routine.^37^ The structures were solved by standard direct methods employing SHELXT^38^ and then refined by full matrix least-squares techniques on *F*^2^ using SHELXL, from the program package SHELX-2018.^38^ As a general method, all atoms different from hydrogen were anisotropically refined, while H atoms were typically included in the structure factor calculations in geometrically idealized positions. However, with the intention of revealing the hydrogen bonding scheme, hydrogen atoms attached to amine nitrogen atoms were located in the corresponding Fourier map. In this case, either they were freely refined or their thermal parameters were derived from their parent atoms.

As commented in the text, it must be noted that the quality of data corresponding to the gadolinium complex **3** was not good enough to be so fully refined as would be desirable. Thus, basically, only atoms different from C and H were anisotropically refined. Despite the remaining electron densities being higher than usual, it must be commented that an attempt to apply SQUEEZE to avoid unassigned electron densities in voids proved useless. Likewise, most of the unassigned charge was found close to the gadolinium and bromine ions. This indicates that significant molecules were not omitted in this crystal structure.

### Powder X-ray Diffraction Studies

The powder diffractograms for **1**–**4**·2H_2_O were recorded on a Philips diffractometer with a control unity type “PW1710”, a vertical goniometer type “PW1820/00”, and a generator type “Enraf Nonius FR590”, operating at 40 kV and 30 mA, using monochromated Cu Kα (λ = 1.5418 Å) radiation. A scan was performed in the range 2 < 2θ < 50° with *t* = 3 s and Δ2θ = 0.02°. LeBail refinement was obtained with the aid of HighScore Plus Version 3.0d.

### Magnetic Measurements

Magnetic susceptibility dc and ac measurements for microcrystalline samples of **1–4**·2H_2_O were carried out with a PPMS Quantum Design susceptometer. The dc magnetic susceptibility data were recorded under a magnetic field of 1000 Oe in the range of 2–300 K. Magnetization measurements at different temperatures (ranging from 2.0 to 7.0 K) were recorded under magnetic fields ranging from 0 to 70 000 Oe. Diamagnetic corrections were estimated from Pascal’s Tables. Alternating current (ac) susceptibility measurements for **1**, **2**, and **4**·2H_2_O were performed at zero dc field and at 600 Oe dc field, with an oscillating ac field of 3.5 Oe and ac frequencies ranging from 50 to 10 000 Hz.

### Computational Details

Orca software (version 5.0.3) was employed to perform multireference calculations^39^ based on the single-crystal X-ray structures of complexes **1**·5CH_3_C_6_H_5_, **2**·2H_2_O·0.75THF, and **4**·2H_2_O·2THF. The fragment approach, where one of the metals is substituted by the close shell La^3+^ ion, was employed to study independently each metal in each dinuclear compound. Due to the symmetry of the molecule, only the results for one of the metals is shown because they were identical.

Due to the large ionic character of the Ln–O/N/F bonds, the inclusion of the dynamic correlation contributions is not necessary. The def2-TZVP basis set was used.^40,41^ For the Dy, a (9,7) active space was employed and 21 sextets, 128 quadruplets, and 98 doublets were considered. In the case of Ho, a (10,7) active space was considered and 35 sextets, 210 quadruplets, and 196 doublets were included. The Single_Aniso and Poly_Aniso^42^ stand-along utilities, distributed with Orca 5.0.3, were employed to evaluate the magnetic properties of the individual fragments and the simulation of the anisotropic exchange interactions. The energy barrier was evaluated with the probability of transition between two different states of the molecules using the matrix elements of the transition magnetic moments, which have been calculated as proposed by the golden Fermi rule, as the integral between the two involved states using a magnetic moment operator.^43^

For the calculation of the exchange coupling constant of complex **3**, DFT calculations were performed with the all-electron FHI-aims computer code using a numerical local orbital basis set (tight basis set in such a computer code).^44^ This approach allows for full-potential calculations at a low computational cost without using any a priori approximations for the potential, such as pseudopotentials or frozen cores. The calculations were performed using the single-crystal X-ray structures of **3** and the hybrid B3LYP exchange–correlation functional.^45^ The calculation of the *J* coupling was performed using the nonprojected option.^46^

## Supporting Information Available

Figures S1−S9 and Tables S1−S9 (PDF)
