# Supplementary material for: Dinuclear Fluoride Single-Bridged Lanthanoid Complexes as Molecule Magnets: Unprecedented Coupling Constant in a Fluoride-Bridged Gadolinium Compound
Source: Inorg Chem. 2022 Jun 23;61(26):9946–59. doi: 10.1021/acs.inorgchem.2c00773 (PMC9275779; doi:10.1021/acs.inorgchem.2c00773)
Supplement: Supplementary file 1 — ic2c00773_si_001.pdf [file ic2c00773_si_001.pdf]

Dinuclear fluoride-single bridged lanthanoid complexes as molecule magnets: unprecedented coupling constant in a fluoride bridged gadolinium compound

Julio Corredoira-Vázquez,[§] Cristina González-Barreira,[§] Matilde Fondo,[§,] Ana M. García-Deibe,[§] Jesús Sanmartín-Matalobos,[§] Silvia Gómez-Coca, [ξ] Eliseo Ruiz,[ξ] and Enrique Colacio[£]*

[§] Departamento de Química Inorgánica, Facultade de Química, Universidade de Santiago de Compostela, Campus Vida, 15782 Santiago de Compostela, Spain.

[ξ] Departament de Química Inorgànica i Orgànica, and Institut de Química Teórica i Computacional, Universitat de Barcelona, 08028 Barcelona, Spain

[£] Departamento de Química Inorgánica, Facultad de Ciencias, Universidad de Granada, Avda Fuentenueva s/n, 18071 Granada, Spain

Table S1. Main bond distances and angles for **1**·5$CH_3C_6H_5$, **2**·2H_2O 0.75THF, **3**, and **4**·2H_2O·2THF page S3

Table S2. SHAPE v2.1. Continuous shape measures calculation (c) 2013 Electronic Structure Group, Universitat de Barcelona page S4

Table S3. Calculated energies (in cm^{-1}) of the states for each individual fragment before the inclusion of the spin-orbit effect for the Ln_2 compounds. And the figure of merit (E2-E1)/E1 for the Dy_2 compounds. page S5

Table S4. Calculated energies (in cm^{-1}) of the 8 lowest Kramers doublets for each individual fragment after the RASSI step for the Dy_2 compounds. page S5

Table S5. Calculated energies (in cm^{-1}) of the 17 lowest states for each individual fragment after the RASSI step for the Ho_2 compounds. page S6

Table S6. Calculated low lying spectrum including the exchange/dipolar coupling for **1**.1. Projected magnetic moment, energy and transition probabilities of the first two doublets to the other 10 lowest doublets. page S6

Table S7. Calculated low lying spectrum including the exchange/dipolar coupling for **1**.2. Projected magnetic moment, energy and transition probabilities of the first two doublets to the other 10 lowest doublets. page S7

Table S8. Calculated low lying spectrum including the exchange/dipolar coupling for **4**. Projected magnetic moment, energy and transition probabilities of the first two doublets to the other 10 lowest doublets. page S7

Table S9. Crystal data and structure refinement for **1**·$5CH_3C_6H_5$-**4**·$2H_2O$·2THF page S8

Figure S1. Comparison of X-powder diffractogram for the crystalline samples of a) **1** (blue); b) **2** (pink); c) **3** (maroon); d) **4**·$2H_2O$ (green) with the with the simulated one from single X-ray data. page S9

Figure S2. Ellipsoid (50% probability) diagram for the {[Ho(3NO_2,5Br-$H_3L^{1,1,4}$)]$_2$(μ-F)}$^-$ anion in **2.**1 page S10

Figure S3. Ellipsoid diagram (50% probability) for the {[Gd(3NO_2,5Br-$H_3L^{1,1,4}$)]$_2$(μ-F)}$^-$ anion in **3.**1. page S10

Figure S4. $\chi_M T$ *vs* T for **2**. Inset: $M/N\mu_B$ *vs* H. page S11

Figure S5. Temperature dependence at different frequencies for χ'_M (left), and χ''_M(right) for **4**·$2H_2O$ in H_{dc} = 0. page S11

Figure S6. Cole–Cole plot for **4**·$2H_2O$ in zero field. page S11

Figure S7. Left) frequency dependence of χ''_M for **4**·$2H_2O$ at 2.5 K under different magnetic fields; right) dependence of the relaxation time with the field. page S12

Figure S8. Temperature dependence of χ''_M for **1** (left), and **4**·$2H_2O$ (right) at different frequencies in H_{dc} = 600 Oe. page S12

Figure S9. Cole–Cole plot for **1** (top), and **4**·$2H_2O$ (bottom) in H_{dc} = 600 Oe page S12

Table S1. Main bond distances and angles for **1**·5$CH_3C_6H_5$, **2**·2H_2O 0.75THF, **3**, and **4**·2H_2O·2THF.

	**1**·5$CH_3C_6H_5$		**2**·2H_2O·0.75THF		**3**		**4**·2H_2O·2THF
	**1**.1 (X = 1)	**1**.2 (X = 2)	**2**.1 (X = 1)	**2**.2 (X = 2)	**3**.1 (X = 1)	**3**.2 (X = 2)	X =1
LnX-OX1	2.298(3)	2.249(3)	2.238(3)	2.274(3)	2.307(10)	2.264(10)	2.237(4)
LnX-OX2	2.298(3)	2.290(3)	2.299(4) .	2.297(4)	2.319(12)	2.323(10)	2.331(4)
LnX-OX3	2.271(3)	2.315(3)	2.289(4)	2.306(4)	2.323(11)	2.303(12)	2.307(4)
LnX-NX1	2.635(3)	2.646(3)	2.641(4)	2.612(4)	2.650(13)	2.661(12)	2.540(5)
LnX-NX2	2.558(4)	2.539(3)	2.518(4)	2.550(4)	2.581(13)	2.558(12)	2.585(5)
LnX-NX3	2.551(4)	2.524(4)	2.514(4)	2.504(4)	2.571(13)	2.587(13)	2.555(5)
LnX-NX4	2.543(3)	2.552(3)	2.534(4)	2.543(4)	2.614(13)	2.571(12)	2.563(5)
LnX-FX	2.2764(3)	2.2717(3)	2.2640(4)	2.2605(3)	2.2795(7)	2.2773(8)	2.1943(5)
LnX ··· LnX	4.5528(6)	4.5434(8)	4.5282(7)	4.5210(6)	4.559(1)	4.555(1)	4.3709(8)
OX3-LnX-NX1	75.99(10)	77.52(11)	75.14(12)	77.90(13)	78.5(4)	75.8(4)	67.74(15)
NX3-LnX-NX4	68.92(12)	68.62(12)	69.59(14)	68.81(15)	68.1(4)	69.4(4)	68.11(15)
OX1-LnX-NX2	138.90(10)	138.56(11)	139.29(13)	139.13(14)	137.6(4)	139.1(4)	145.74(15)
OX1-LnX-NX3	153.60(10)	152.14(11)	151.53(14)	151.45(14)	153.2(4)	152.6(4)	144.94(14)
OX3-LnX-FX	138.92(7)	140.76(7)	140.37(9)	141.79(9)	140.8(2)	139.7(3)	142.68(12)
LnX-FX-LnX	180.0	180.0	180.0	180.0	180.0	180.0	169.8(2)

Table S2. SHAPE v2.1. Continuous Shape Measures calculation (c) 2013 Electronic Structure Group, Universitat de Barcelona.

Geometries Coordination number 8

ETBPY-8	13 D3h	Elongated trigonal bipyramid
TT-8	12 Td	Triakis tetrahedron
JSD-8	11 D2d	Snub diphenoid J84
BTPR-8	10 C2v	Biaugmented trigonal prism
JBTPR-8	9 C2v	Biaugmented trigonal prism J50
JETBPY-8	8 D3h	Johnson elongated triangular bipyramid J14
JGBF-8	7 D2d	Johnson gyrobifastigium J26
TDD-8	6 D2d	Triangular dodecahedron
SAPR-8	5 D4d	Square antiprism
CU-8	4 Oh	Cube
HBPY-8	3 D6h	Hexagonal bipyramid
HPY-8	2 C7v	Heptagonal pyramid
OP-8	1 D8h	Octagon

Bu$_4$N[Dy(3NO$_2$,5Br-H$_3$L^{1,1,4})$_2$F]·5CH$_3$C$_6$H$_5$ (**1**·5CH$_3$C$_6$H$_5$)

1.1

Structure [ML8]	ETBPY-8	TT-8	JSD-8	**BTPR-8**	JBTPR-8	JETBPY-8
	24.729,	12.634,	2.597,	**2.287**,	2.516,	26.172
JGBF-8	**TDD-8**	SAPR-8	CU-8	HBPY-8	HPY-8	OP-8
12.106,	**1.401**,	2.563,	12.038,	15.621,	23.651,	31.567

1.2

Structure [ML8]	ETBPY-8	TT-8	JSD-8	**BTPR-8**	JBTPR-8	JETBPY-8
	24.402,	12.746,	2.425,	**2.306**,	2.471,	25.688,
JGBF-8	**TDD-8**	SAPR-8	CU-8	HBPY-8	HPY-8	OP-8
11.938,	**1.252**,	2.455,	12.042,	15.403,	23.028,	31.836

Bu$_4$N[Ho(3NO$_2$,5Br-H$_3$L^{1,1,4})$_2$F]·0.75C$_4$H$_4$O·2H$_2$O (**2**·2H$_2$O·0.75THF)

2.1

Structure [ML8]	ETBPY-8	TT-8	**JSD-8**	BTPR-8	JBTPR-8	JETBPY-8
	25.294,	12.204,	**2.301**,	2.564,	2.727,	26.818,,
JGBF-8	**TDD-8**	SAPR-8	CU-8	HBPY-8	HPY-8	OP-8
12.009,	**1.020**,	3.171,	11.800,	16.166,	23.821,	31.169,

2.2

Structure [ML8]	ETBPY-8	TT-8	JSD-8	BTPR-8	JBTPR-8	JETBPY-8
	24.948,	12.394,	2.477,	2.444,	2.689,	26.278,
JGBF-8	**TDD-8**	**SAPR-8**	CU-8	HBPY-8	HPY-8	OP-8
11.967,	**1.159**,	**2.344**,	11.977,	15.584,	22.844,	31.464

Bu$_4$N[Gd(3NO$_2$,5Br-H$_3$L^{1,1,4})$_2$F] (**3**)

3.1

Structure [ML8]	ETBPY-8	TT-8	JSD-8	**BTPR-8**	JBTPR-8	JETBPY-8
	24.155,	12.439,	2.633,	**2.488**,	2.772,	25.401,
JGBF-8	**TDD-8**	SAPR-8	CU-8	HBPY-8	HPY-8	OP-8
11.890,	**1.418**,	2.475,	11.874,	15.064,	22.591,	31.647

3.2

Structure [ML8]	ETBPY-8	TT-8	JSD-8	BTPR-8	JBTPR-8	JETBPY-8
	25.002,	12.391,	**2.471**,	2.605,	2.831,	26.575,
JGBF-8	**TDD-8**	SAPR-8	CU-8	HBPY-8	HPY-8	OP-8
11.980,	**1.139**,	3.294,	11.972,	16.024,	23.696,	31.106

$Bu_4N\{[Dy(3Br,5Cl\text{-}H_3L^{1,2,4})]_2(\mu\text{-}F)\}\cdot 2H_2O\cdot 2THF$ (**4**$\cdot 2H_2O\cdot 2THF$)

Structure [ML8]	ETBPY-8	TT-8	JSD-8	BTPR-8	JBTPR-8	JETBPY-8
	24.362,	11.809,	2.974,	**1.371**,	**1.341**,	27.947
JGBF-8	**TDD-8**	SAPR-8	CU-8	HBPY-8	HPY-8	OP-8
12.350,	**1.321**,	1.856,	11.265,	16.169,	24.126,	30.397

Table S3. Calculated energies (in cm^{-1}) of the states for each individual fragment before the inclusion of the spin-orbit effect for the Ln_2 compounds. And the figure of merit (E2-E1)/E1 for the Dy_2 compounds.

State	1.1 (Dy1)	1.2 (Dy2)	4 (Dy1)	2.1 (Ho1)	2.2 (Ho2)
1	0	0	0	0	0
2	20.6	15.9	14	6.3	14.3
3	106.3	127	146	61.6	58
4	176.2	214.7	212.3	110.8	99.4
5	215.4	226.8	222.2	125.9	131.5
6	243.8	241.6	275	154.7	161.8
7	267.8	263.4	296	171.6	173.8
8	287.8	305.3	333.9	193.3	188.4
(E2-E1)/E1	4.16	6.99	9.43		

Table S4. Calculated energies (in cm^{-1}) of the 8 lowest Kramers doublets for each individual fragment after the RASSI step for the Dy_2 compounds.

KDs	1.1 (Dy1)	1.2 (Dy2)	4 (Dy1)
1	0	0	0
2	95.8	102.19	100.07
3	107.01	158.06	155.71
4	151.62	179.24	175.09
5	193.57	208.37	211.97
6	216.01	238.01	256.51
7	282.99	324.07	294.12
8	339.11	386.02	389.01

Table S5. Calculated energies (in cm^{-1}) of the 17 lowest states for each individual fragment after the RASSI step for the Ho_2 compounds.

State	2.1 (Ho1)	2.2 (Ho2)
1	0	0
2	3.58	6.5
3	13.72	17.84
4	21.62	22.40
5	43.09	34.24
6	57.19	59.76
7	74.44	78.60
8	90.28	92.50
9	107.59	100.76
10	130.94	139.37
11	165.79	153.54
12	192.66	193.55
13	210.48	198.26
14	227.38	223.03
15	234.05	227.45
16	239.57	244.15
17	246.20	246.28

Table S6. Calculated low lying spectrum including the exchange/dipolar coupling for **1**.1. Projected magnetic moment, energy and transition probabilities of the first two doublets to the other 10 lowest doublets.

M	Energy (cm^{-1})	Tunneling	Orbach (1 -> i)	Orbach (2 -> i)
0.000487	7.36E-05	3.42E-12		
1.56	1.17	6.17E-11	1.27E-01	
1.09	96.26	3.02E-07	1.24E+00	2.28E-01
1.21	96.28	1.75E-07	1.91E-01	3.09E-01
0.65	96.45	2.20E-07	5.01E-01	1.11E-01
1.09	96.53	9.00E-08	4.00E-01	2.14E+00
0.25	107.46	1.34E-07	9.34E-01	4.43E-02
0.27	107.48	9.73E-08	1.51E-01	2.68E-02
1.27	107.68	2.69E-08	3.75E-01	5.19E-02
1.20	107.71	3.99E-08	1.25E-01	2.06E-02

Table S7. Calculated low lying spectrum including the exchange/dipolar coupling for **1**.2. Projected magnetic moment, energy and transition probabilities of the first two doublets to the other 10 lowest doublets.

M	Energy (cm^{-1})	Tunneling	Orbach (1 -> i)	Orbach (2 -> i)
0.0004	5.52E-05	7.30E-12		
14.63	1.230148	5.19E-12	9.50E-02	
3.80	102.5803	4.43E-09	1.78E-01	9.96E-02
3.78	102.6233	9.02E-09	1.93E+00	1.18E-01
10.08	102.901	1.17E-08	3.28E-01	4.19E-02
10.17	102.9984	7.52E-09	3.11E-01	1.05E-02
6.21	158.5129	3.57E-06	5.15E-02	1.17E-01
6.22	158.5323	5.44E-06	3.86E-01	9.72E-02
6.50	158.8277	2.75E-06	3.46E-02	1.25E-01
6.50	158.8496	3.00E-06	6.19E-01	1.33E-01

Table S8. Calculated low lying spectrum including the exchange/dipolar coupling for **4**. Projected magnetic moment, energy and transition probabilities of the first two doublets to the other 10 lowest doublets.

M	Energy (cm^{-1})	Tunneling	Orbach (1 -> i)	Orbach (2 -> i)
5.66	9.81E-05	3.89E-12		
0.0055	1.55	1.07E-09	4.60E-02	
4.82	100.20	3.15E-10	2.71E-03	1.58E-02
4.84	100.43	1.27E-10	2.68E-03	6.71E-02
0.27	101.24	1.17E-08	5.32E-02	9.13E-04
0.23	101.92	7.25E-09	5.02E-02	3.14E+00
3.20	155.45	2.65E-09	2.01E-01	2.12E-01
3.20	156.08	4.21E-07	1.93E-01	1.69E-03
8.78	157.49	6.72E-09	1.51E-03	2.24E-03
8.75	158.09	4.96E-07	1.53E-03	1.92E-01

Table S9. Crystal data and structure refinement for **1**·$5CH_3C_6H_5$-**4**·$2H_2O$·2THF

	**1**·$5CH_3C_6H_5$	**2**·$2H_2O$·0.75THF	**3**	**4**·$2H_2O$·2THF
Empirical formula	$C_{105}H_{130}Br_6Dy_2FN_{15}O_{18}$	$C_{73}H_{96}Br_6FHo_2N_{15}O_{20.75}$	$C_{70}H_{90}Br_6FGd_2N_{15}O_{18}$	$C_{78}H_{106}Br_6Cl_6Dy_2FN_9O_{10}$
Molecular weight	2713.69	2343.96	2242.52	2365.87
Crystal system	Triclinic	Triclinic	Triclinic	Orthorhombic
Space group	P-1	P-1	P-1	Pnna
Wavelength (Å)	0.71073	0.71073	0.71073	0.71073
Crystal size (mm^3)	0.110 x 0.100 x 0.070	0.090 x 0.080 x 0.020	0.272 x 0.039 x 0.009	0.17 x 0.06 x 0.02
Color, shape	Prism, yellow	Plate, yellow	Needle, orange	Needle, colourless
T (K)	100(2)	100(2)	100(2)	100(2)
a (Å)	14.5642(16)	14.8933(17)	14.8139(15)	19.509(2)
b (Å)	16.3913(18)	17.654(2)	17.4763(18)	25.123(2)
c (Å)	23.535(3)	19.350(2)	19.3926(19)	18.5980(17)
α (º)	80.078(2)	67.896(4)	68.033(3)	90
β (º)	85.103(2)	68.863(4)	69.415(3)	90
γ (º)	80.9430(10)	80.623(4)	81.435(3)	90
Volume ($Å^3$)	5455.5(10)	4394.3(9)	4357.9(8)	9115.4(16)
Z	2	2	2	4
Absorpt. coef. (mm^{-1})	3.630	4.593	4.332	4.493
Reflections collected	134302	109867	293404	203084
Independent reflections	22309 [R_{int} = 0.0835]	17970 [R_{int} = 0.0584]	17830 [R_{int} = 0.1714]	11310 [R(int) = 0.1414]
Data / restraints / param.	22309 / 6 / 1324	17970 / 70 / 1129	17830 / 0 / 572	11310 / 0 / 602
Final R indices [$I>2\sigma(I)$]	R_1 = 0.0387;wR_2 = 0.0763	R_1 = 0.0410; wR_2 = 0.0907	R_1 = 0.1263; wR_2 = 0.2591	R_1 = 0.0497; wR_2 = 0.1081
R indices (all data)	R_1 = 0.0646;wR_2 = 0.0850	R_1 = 0.0635; wR_2 = 0.1017	R_1 = 0.1415; wR_2 = 0.2672	R_1 = 0.0877;wR_2 = 0.1278

a)

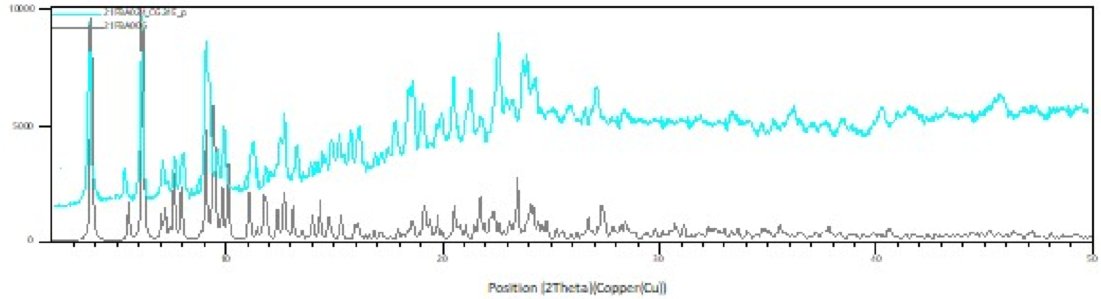

b)

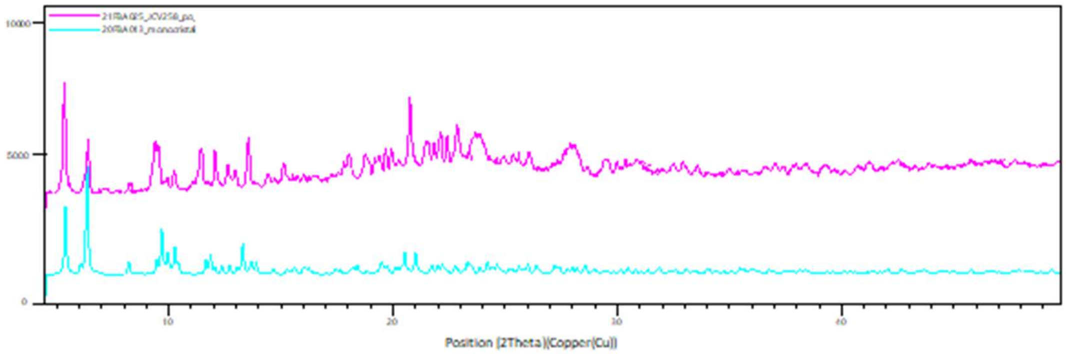

c)

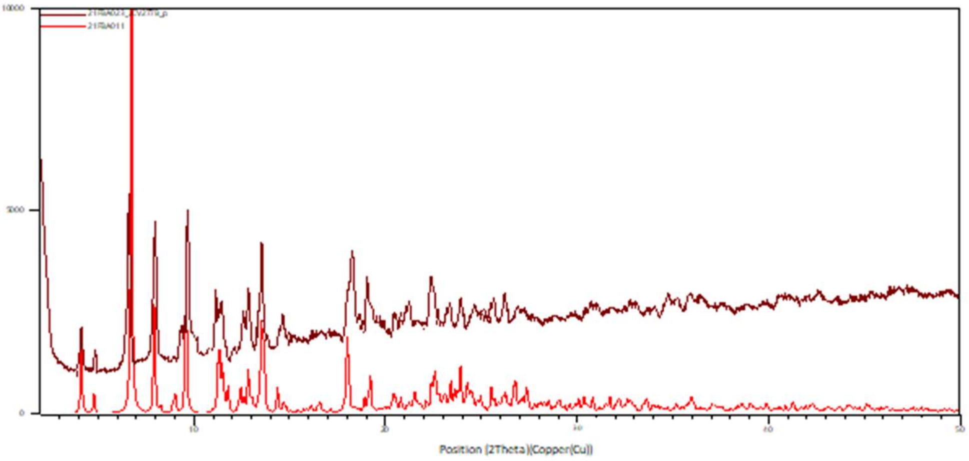

d)

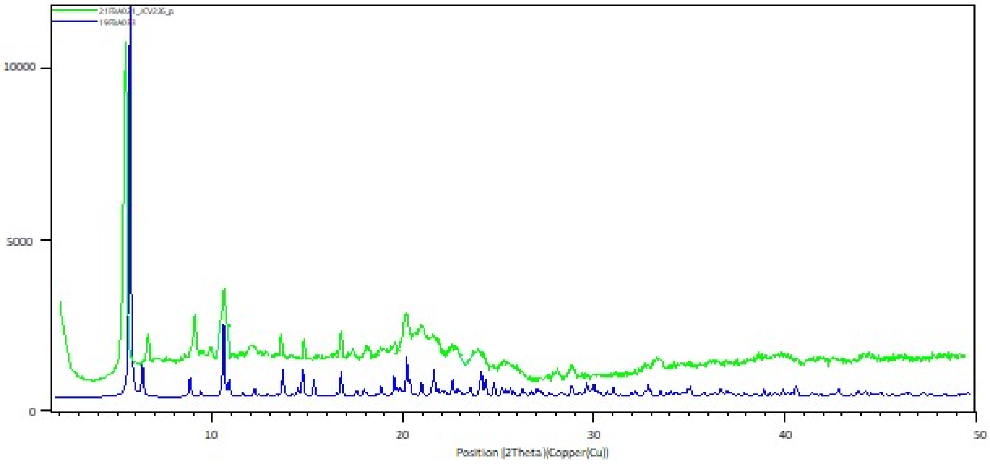

Figure S1. Comparison of X-powder diffractogram for the crystalline samples of a) **1** (blue); b) **2** (pink); c) **3** (maroon); d) **4**·$2H_2O$ (green) with the with the simulated one from single X-ray data.

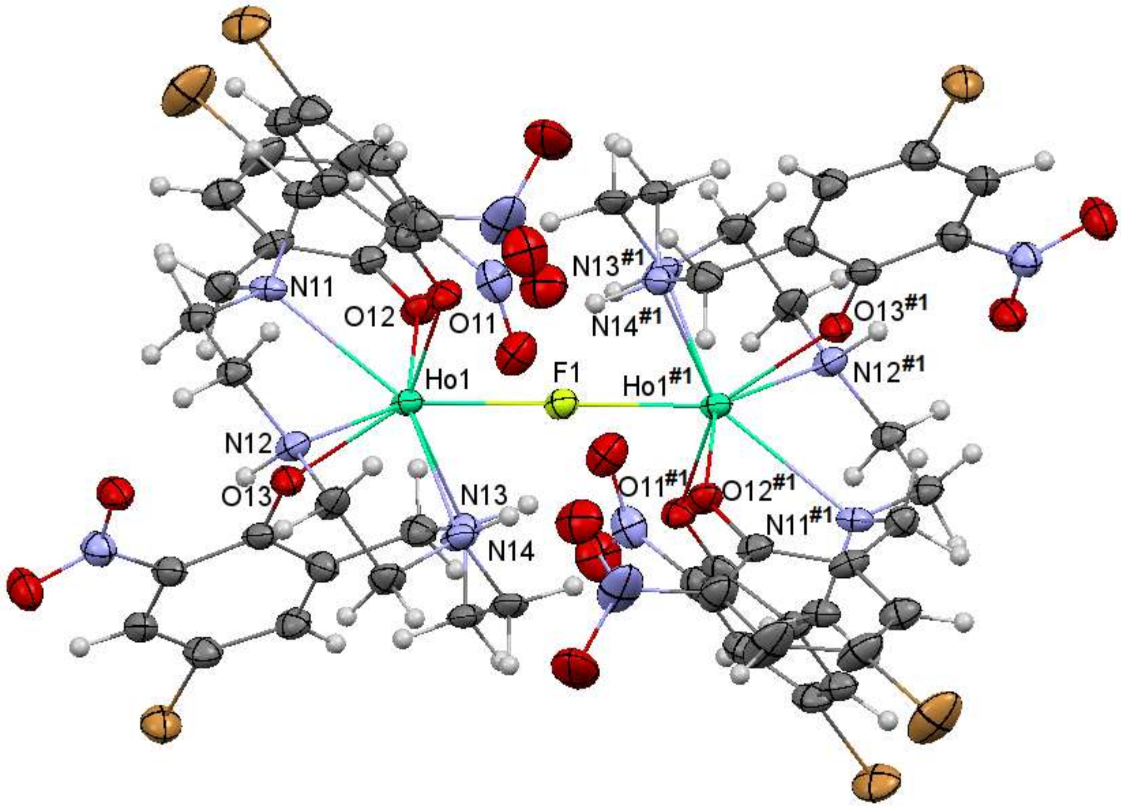

Figure S2. Ellipsoid diagram (50% probability) for the $\{[Ho(3NO_2,5Br\text{-}H_3L^{1,1,4})]_2(\mu\text{-}F)\}^-$ anion in **2.**1.

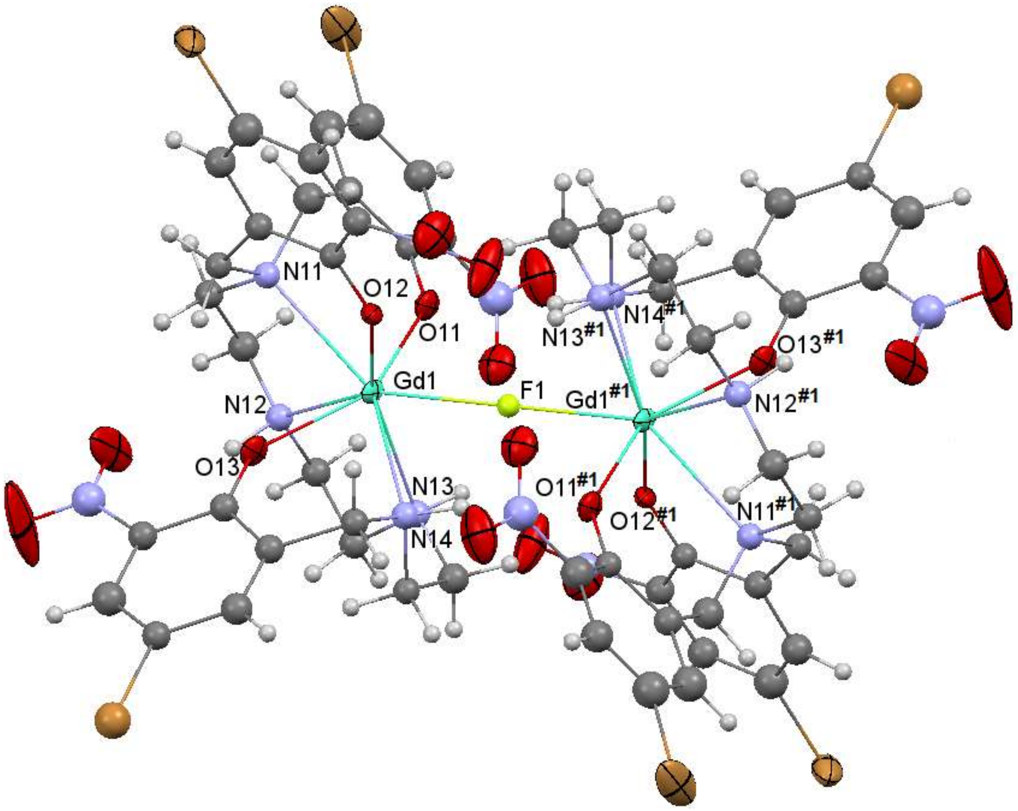

Figure S3. Ellipsoid diagram (50% probability) for the $\{[Gd(3NO_2,5Br\text{-}H_3L^{1,1,4})]_2(\mu\text{-}F)\}^-$ anion in **3.**1.

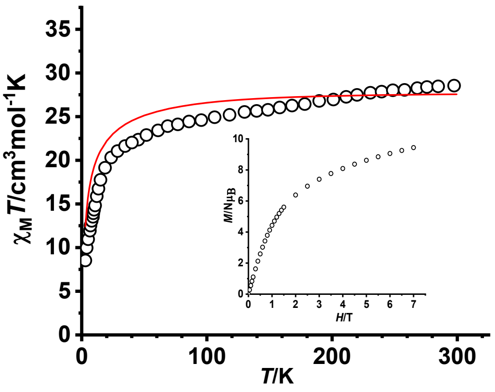

Figure S4. $\chi_M T$ *vs* T for **2**. Inset: $M/N\mu_B$ *vs* H at 3 K. The solid line represents the theoretical data obtained from *ab initio* calculations.

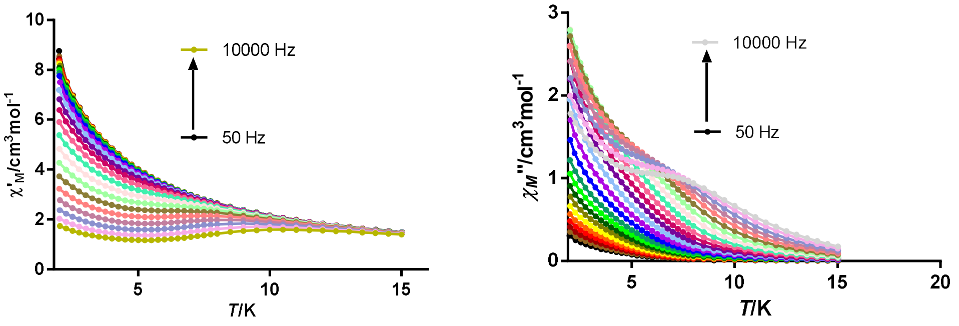

Figure S5. Temperature dependence at different frequencies for χ'_M (left), and χ''_M (right) for **4**·$2H_2O$ in H_{dc} = 0.

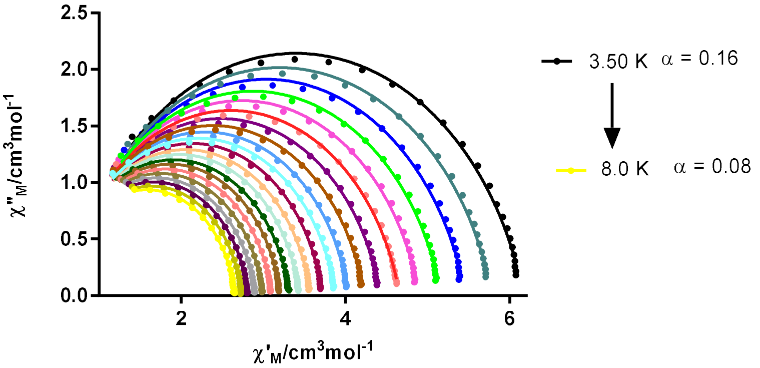

Figure S6. Cole–Cole plot for **4**·$2H_2O$ in zero field.

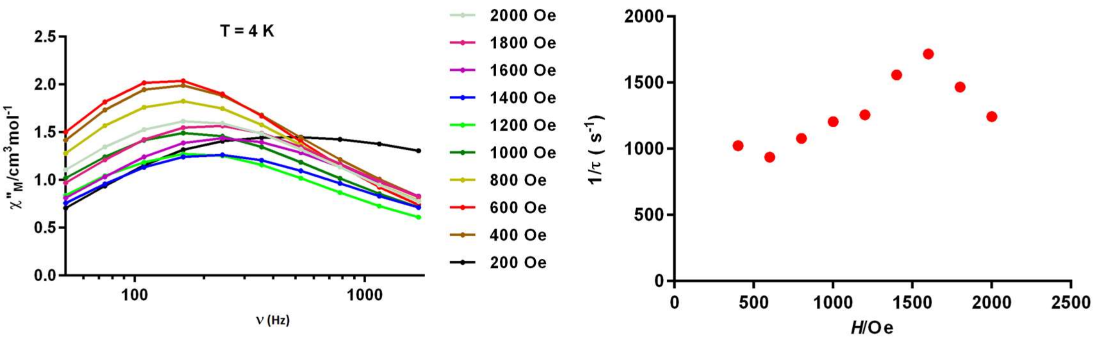

Figure S7. Left) frequency dependence of χ''_M for **4**·$2H_2O$ at 2.5 K under different magnetic fields; right) dependence of the relaxation time with the field.

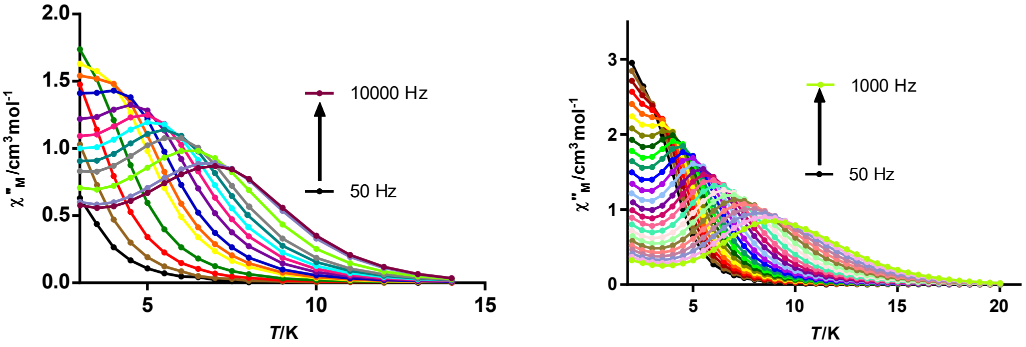

Figure S8. Temperature dependence of χ''_M for **1** (left), and **4**·$2H_2O$ (right) at different frequencies in H_{dc} = 600 Oe.

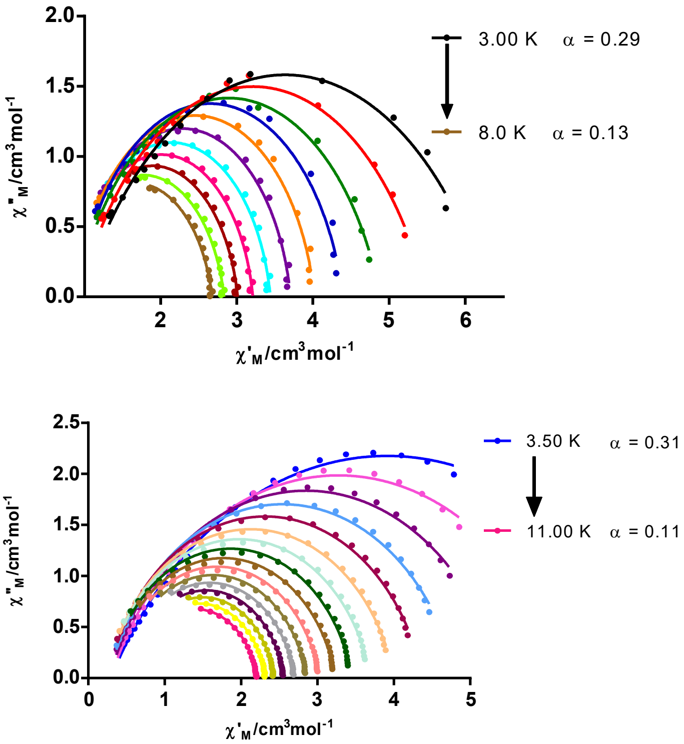

Figure S9. Cole–Cole plot for **1** (top), and **4**·$2H_2O$ (bottom) in H_{dc} = 600 Oe.
